# Supplementary material for: Aphid Herbivory Drives Asymmetry in Carbon for Nutrient Exchange between Plants and an Arbuscular Mycorrhizal Fungus
Source: Curr Biol. 2020 May 18;30(10):1801–1808.e5. doi: 10.1016/j.cub.2020.02.087 (PMC7237887; doi:10.1016/j.cub.2020.02.087)
Supplement: Document S1. Figures S1–S5 and Tables S1 and S2 [file mmc1.pdf]

Current Biology, Volume *30*

Supplemental Information

Aphid Herbivory Drives Asymmetry in Carbon for Nutrient Exchange between Plants and an Arbuscular Mycorrhizal Fungus

Michael D. Charters, Steven M. Sait, and Katie J. Field

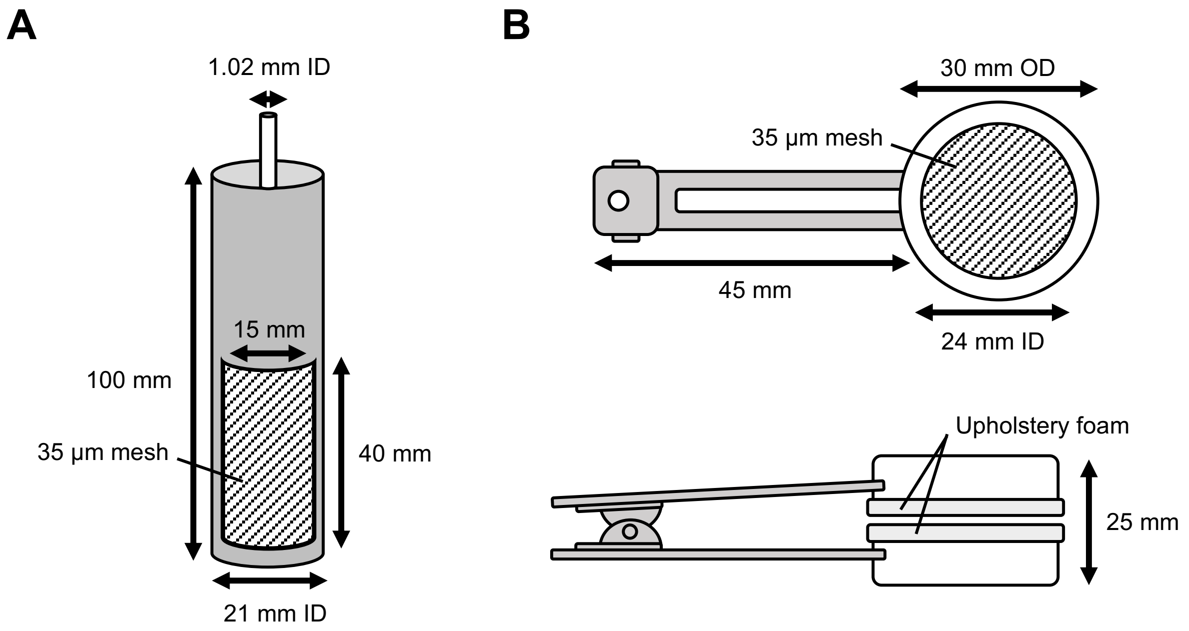

Figure S1. Schematic diagrams of PVC cores and insect clips cages. 'ID' and 'OD' refer to inner and outer diameter measurements. Related to Figure 1 and STAR METHODS.
(A) Dimensions of the three PVC cores added to the substrate of each pot. Two windows were machine-cut into opposite faces of the core, and covered with 35 µm nylon mesh using an acrylic adhesive. Mesh apertures permitted in-growth of AM fungal hyphae but not plant roots. A silicone capillary tube was perforated and attached centrally. This acted as a conduit for the introduction of ^{33}P to the two substrate-filled cores. The third core contained glass wool and had a rubber septum fitted on top. **(B)** Dimensions of the insect clip cages positioned on the third leaf of the primary tiller of plants. Cages were constructed using machine-cut transparent acrylic tubing and double prong curl setting clips. Upholstery foam and 35 µm nylon mesh were attached to the inside and outside faces of the cage using an acrylic adhesive, which limited damage to the leaf surface and kept aphids confined.

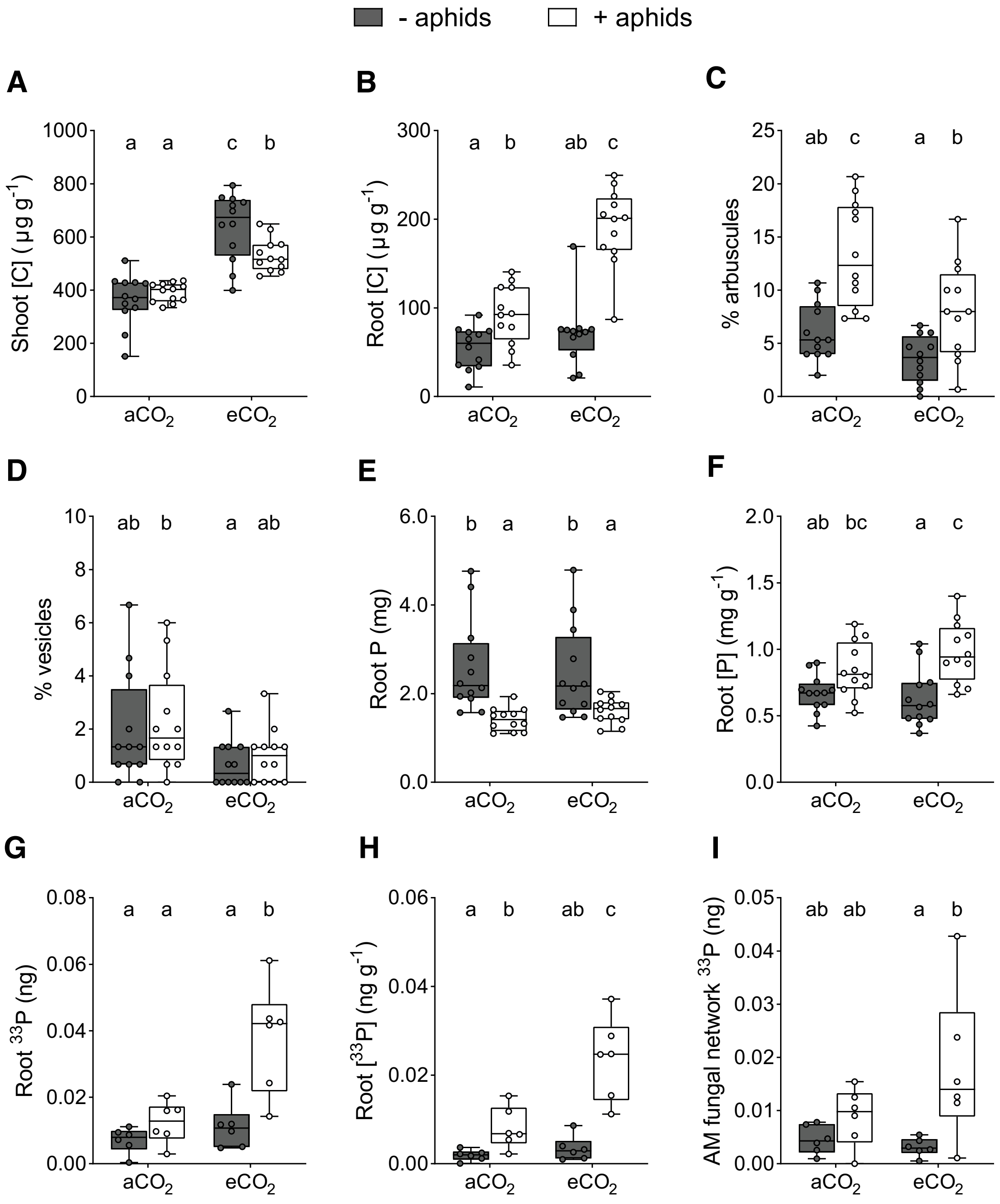

Figure S2. Supplementary data for traits in plants not exposed (gray boxes) or exposed (white boxes) to aphid herbivores at ambient and elevated atmospheric [CO_2]. Related to Figures 2, 3, and 5.

(A) Shoot C concentration. **(B)** Root C concentration. **(C)** % arbuscular colonization. **(D)** % vesicular colonization. **(E)** Root P content. **(F)** Root P concentration. **(G)** Root ^{33}P content. **(H)** Root ^{33}P concentration. **(I)** ^{33}P in the AM fungal hyphal network. Boxplots extend from the first to the third quartile, with the middle line representing median values (n = 12, except for G, H, and I where n = 6). Whiskers are drawn to the minimum and maximum data points (open or closed markers). Different letters denote significant differences between treatment means (where $p < 0.05$, GLM+Tukey HSD tests).

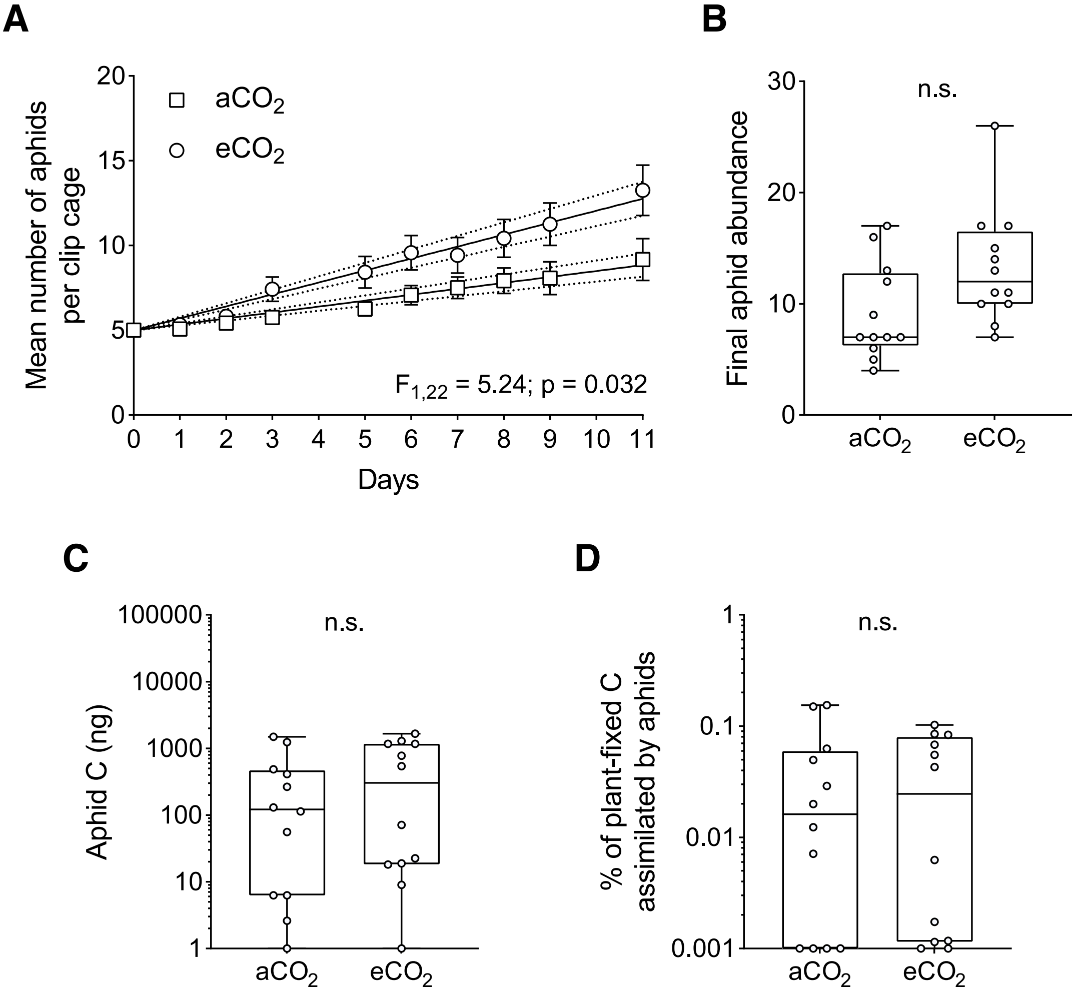

Figure S3. Effect of atmospheric [CO_2] on wheat-feeding aphids (*Rhopalosiphum padi*). Related to Figure 4 and STAR METHODS.

(A) Aphid population growth rates in insect clip cages on 8-week old plants grown at aCO_2 (440 ppm; circular markers) and eCO_2 (800 ppm; square markers) during the 12-day isotope labelling period (n = 12; mean ± SE). Dashed lines represent 95% confidence intervals. **(B)** Final aphid abundance inside insect clip cages prior to the $^{14}CO_2$ pulse. **(C)** Assimilation of recently fixed plant C by aphids (log scale). **(D)** % of plant-fixed C assimilated by aphids (log scale). Boxplots extend from the first to the third quartile, with the middle line representing median values (n = 12). Whiskers are drawn to the minimum and maximum data points (open markers). 'n.s.' denotes no significant difference between treatments, as final aphid abundance ($F_{1,22} = 3.73$; $p = 0.066$), aphid C ($F_{1,22} = 0.23$; $p = 0.636$), and the % of plant-fixed C assimilated by aphids ($F_{1,22} = 0.01$; $p = 0.961$) were equivalent at aCO_2 and eCO_2.

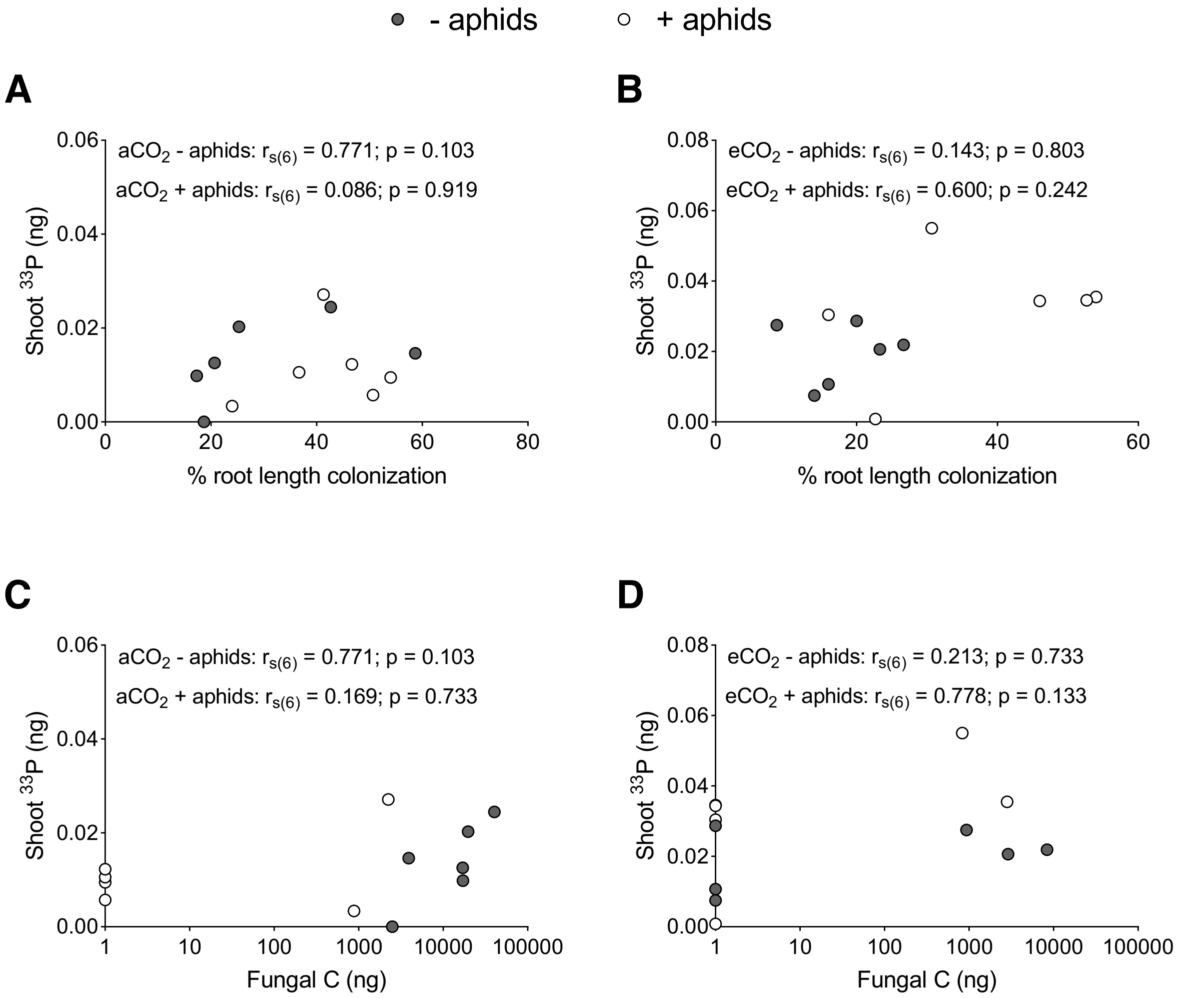

Figure S4. Mycorrhizal uptake of ^{33}P as a function of AM abundance in roots of wheat or transfer of recently-fixed plant C to the AM fungus. Related to Figures 3, 4, and 5.

(A-B) Relationship between AM fungal-acquired shoot ^{33}P and % root length colonization in plants not exposed (gray markers) and exposed (white markers) to aphid herbivores at ambient (A) and elevated (B) atmospheric [CO_2]. Associations analyzed using Spearman's rank correlation coefficient.

(C-D) Relationship between AM fungal-acquired shoot ^{33}P and plant C transfer to the AM fungus in plants not exposed (gray markers) and exposed (white markers) to aphid herbivores at ambient (C) and elevated (D) atmospheric [CO_2]. Associations analyzed using Spearman's rank correlation coefficient.

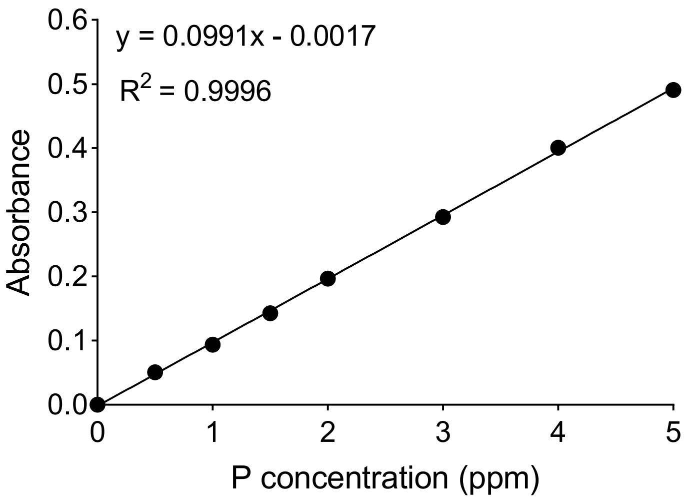

Figure S5. Calibration curve for total P determination of plant material. Related to Figure 5 and STAR METHODS.

Response variable	Factor	F-value	df	p-value
Shoot [C]	Aphids	2.40	1,44	0.128
	[CO_2]	67.36	1,44	**<0.001**
	Aphids * [CO_2]	7.13	1,44	**0.011**
Root [C]	Aphids	36.61	1,44	**<0.001**
	[CO_2]	13.92	1,44	**<0.001**
	Aphids * [CO_2]	3.37	1,44	0.073
% arbuscules	Aphids	27.03	1,44	**<0.001**
	[CO_2]	13.64	1,44	**<0.001**
	Aphids * [CO_2]	0.26	1,44	0.610
% vesicles	Aphids	1.10	1,44	0.300
	[CO_2]	8.91	1,44	**0.005**
	Aphids * [CO_2]	0.01	1,44	0.915
Root P content	Aphids	28.72	1,44	**<0.001**
	[CO_2]	0.16	1,44	0.687
	Aphids * [CO_2]	1.21	1,44	0.278
Root [P]	Aphids	21.14	1,44	**<0.001**
	[CO_2]	0.67	1,44	0.416
	Aphids * [CO_2]	2.43	1,44	0.126
Root ^{33}P content	Aphids	16.28	1,20	**<0.001**
	[CO_2]	13.38	1,20	**0.002**
	Aphids * [CO_2]	4.29	1,20	0.051
Root [^{33}P]	Aphids	47.88	1,20	**<0.001**
	[CO_2]	15.02	1,20	**<0.001**
	Aphids * [CO_2]	6.04	1,20	**0.023**
AM fungal network ^{33}P	Aphids	7.86	1,20	**0.011**
	[CO_2]	0.74	1,20	0.401
	Aphids * [CO_2]	2.41	1,20	0.136

Table S1. GLM outcomes: main treatment effects (aphid herbivory and atmospheric [CO_2]) and their interaction on response variables measured. Significant p-values ($p < 0.05$) are in bold. Related to Figures 2, 3, 5, and S2.

Response variable	Factor	U_{min}	U_{max}	$n_1 = n_2$	p-value
Fungal C	(i) aCO_2 - aphids * aCO_2 + aphids	22	122	12	**0.002**
	(ii) aCO_2 - aphids * eCO_2 - aphids	56.5	87.5	12	0.367
	(iii) aCO_2 - aphids * eCO_2 + aphids	32	112	12	**0.015**
	(iv) eCO_2 - aphids * eCO_2 + aphids	35	109	12	**0.025**
	(v) aCO_2 + aphids * eCO_2 + aphids	60	84	12	0.362
	(vi) aCO_2 + aphids * eCO_2 - aphids	25	119	12	**0.003**

Table S2. Mann-Whitney U outcomes: main treatment effects (aphid herbivory [i & iv] and atmospheric [CO_2] [ii & v]), and their interaction (iii & vi) on the transfer of recently-fixed plant C to the AM hyphal network in the pot. Significant p-values ($p < 0.05$) are in bold. Related to Figure 4A.
