# Supplementary material for: Aphid Herbivory Drives Asymmetry in Carbon for Nutrient Exchange between Plants and an Arbuscular Mycorrhizal Fungus
Source: Curr Biol. 2020 May 18;30(10):1801–1808.e5. doi: 10.1016/j.cub.2020.02.087 (PMC7237887; doi:10.1016/j.cub.2020.02.087)
Supplement: Document S2. Article plus Supplemental Information [file mmc2.pdf]

Current Biology

Article

Aphid Herbivory Drives Asymmetry in Carbon for Nutrient Exchange between Plants and an Arbuscular Mycorrhizal Fungus

Graphical Abstract

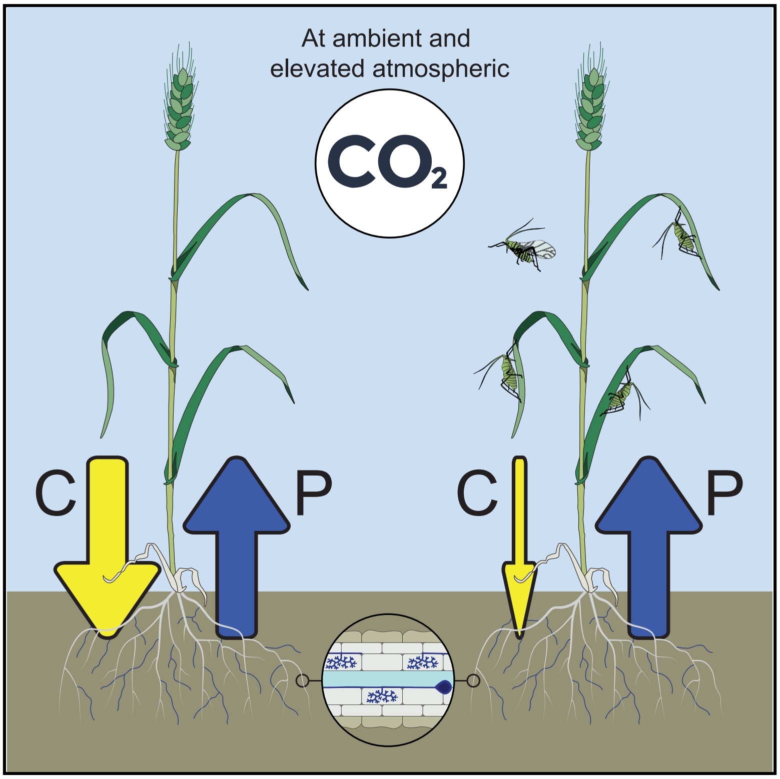

Highlights

- We tested how aphid herbivory and increasing CO_2 affects wheat mycorrhizal function
- Aphids reduced wheat C supply to mycorrhizal fungi. High CO_2 had no effect.
- Transfer of fungal ^{33}P to plant was maintained regardless of aphids or high CO_2
- Mycorrhizal function is context dependent, affected by biotic and abiotic factors

Authors

Michael D. Charters, Steven M. Sait, Katie J. Field

Correspondence

k.field@leeds.ac.uk

In Brief

Little is known about how mycorrhizal function is affected by insect herbivory and environment. Charters et al. show aphids reduce plant C allocation to mycorrhizas, although fungal P transfer to plants is maintained, suggesting high context dependency where resource exchange between symbionts is influenced by interacting biotic and abiotic factors.

Charters et al., 2020, Current Biology *30*, 1801–1808
May 18, 2020 Crown Copyright © 2020 Published by Elsevier Inc.
https://doi.org/10.1016/j.cub.2020.02.087

CellPress

Aphid Herbivory Drives Asymmetry in Carbon for Nutrient Exchange between Plants and an Arbuscular Mycorrhizal Fungus

Michael D. Charters,[1] Steven M. Sait,[1] and Katie J. Field[1,2,*]
[1]School of Biology, Faculty of Biological Sciences, University of Leeds, Leeds LS2 9JT, UK
[2]Lead Contact
*Correspondence: k.field@leeds.ac.uk
https://doi.org/10.1016/j.cub.2020.02.087

SUMMARY

Associations formed between plants and arbuscular mycorrhizal (AM) fungi are characterized by the bi-directional exchange of fungal-acquired soil nutrients for plant-fixed organic carbon compounds. Mycorrhizal-acquired nutrient assimilation by plants may be symmetrically linked to carbon (C) transfer from plant to fungus or governed by sink-source dynamics. Abiotic factors, including atmospheric CO_2 concentration ([CO_2]), can affect the relative cost of resources traded between mutualists, thereby influencing symbiotic function. Whether biotic factors, such as insect herbivores that represent external sinks for plant C, impact mycorrhizal function remains unstudied. By supplying ^{33}P to an AM fungus (*Rhizophagus irregularis*) and $^{14}CO_2$ to wheat, we tested the impact of increasing C sink strength (i.e., aphid herbivory) and increasing C source strength (i.e., elevated [CO_2]) on resource exchange between mycorrhizal symbionts. Allocation of plant C to the AM fungus decreased dramatically following exposure to the bird cherry-oat aphid (*Rhopalosiphum padi*), with high [CO_2] failing to alleviate the aphid-induced decline in plant C allocated to the AM fungus. Mycorrhizal-mediated uptake of ^{33}P by plants was maintained regardless of aphid presence or elevated [CO_2], meaning insect herbivory drove asymmetry in carbon for nutrient exchange between symbionts. Here, we provide direct evidence that external biotic C sinks can limit plant C allocation to an AM fungus without hindering mycorrhizal-acquired nutrient uptake. Our findings highlight the context dependency of resource exchange between plants and AM fungi and suggest biotic factors—individually and in combination with abiotic factors—should be considered as powerful regulators of symbiotic function.

INTRODUCTION

More than 80% of land plants associate with arbuscular mycorrhizal (AM) fungi [1], forming mycorrhizal associations in plants with roots and mycorrhiza-like associations in plants without roots [2]. These intimate symbioses are ancient, dating back to the origins of land plants [3], and are usually considered to be mutualistic. Plants hosting AM fungi gain a number of physiological benefits, including enhanced access to soil nutrients, such as phosphorus (P), via extra-radical fungal hyphae that extend beyond the nutrient depletion zones of host plant roots [4]. AM fungal-derived benefits may also include enhanced plant-pathogen protection [5] and/or improved tolerance against insect herbivores [6] through priming of the host-plant immune system [7].

As obligate biotrophs [8], AM fungi rely exclusively on their plant partners to meet their carbon (C) requirements and, as such, may exert significant C demands on their hosts. AM colonization can increase the C sink strength of roots compared to their non-mycorrhizal counterparts [9], with plant hosts supplying AM fungi with up to 30% of their carbon fixed through photosynthesis [10] as sugars and/or lipids [11]. The relative C sink strength of mycorrhizal roots is largely determined by the C requirements of the fungus [12], combined with abiotic factors, such as the concentration of atmospheric carbon dioxide ([CO_2]). High [CO_2] can increase plant C allocation to mycorrhizal symbionts by up to 25% [13] and, as a consequence, root-internal and root-external abundance of AM fungi [14], likely due to increased photosynthesis and availability of plant C [15]. Thus, [CO_2] can be a powerful environmental variable affecting plant C source strength for AM fungi.

Evidence suggests that the amount of plant C transferred to AM fungi may be tightly regulated by host assimilation of fungal-acquired nutrients [16]. This coordination in resource exchange between symbionts suggests that plants can discriminate between mutualistic mycorrhizal fungal partners [17], withholding plant C from symbionts that do not supply their host with nutrients while preferentially allocating C to more "cooperative" AM fungal isolates [18]. In return, mycorrhizal-mediated nutrient assimilation may be stimulated by plant C allocation to AM fungi [19]. However, resource exchange between mycorrhizal symbionts is not always symmetrically linked [20], being affected by host plant identity [21] and [CO_2] [22], for instance. Despite this context dependency, the influence of biotic and abiotic factors—both individually and in combination—on carbon for nutrient exchange between plants and AM fungi is frequently overlooked.

Insect herbivores represent important external biotic sinks for plant C, directly competing with arbuscular mycorrhizal fungi for plant C resources [23]. Phloem feeders, such as aphids, feed non-destructively on plants by siphoning C-rich sap from phloem sieve tubes [24]. Aphids may further impact the C source

Crown Copyright © 2020 Published by Elsevier Inc.
This is an open access article under the CC BY license (http://creativecommons.org/licenses/by/4.0/).

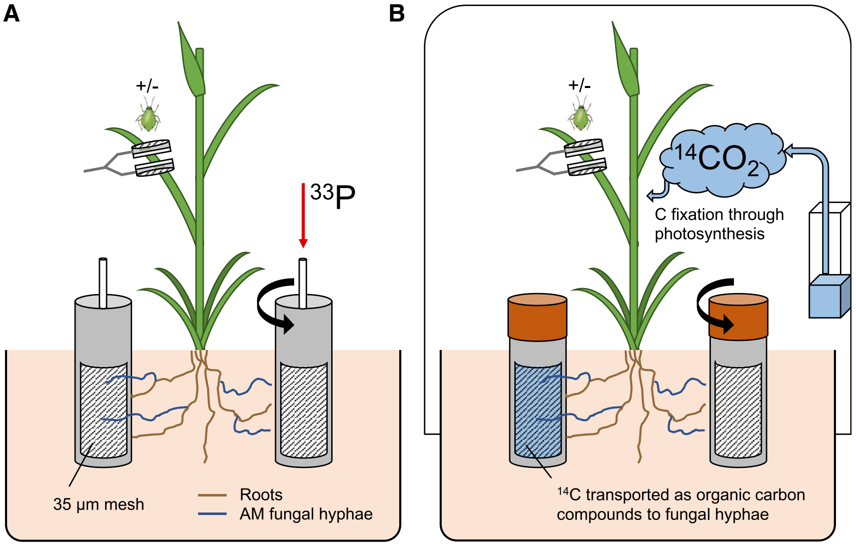

Figure 1. Dual Isotope Tracing Approach for Investigating C Sink-Source Strength Dynamics on Carbon for Nutrient Exchange between Wheat (*Triticum aestivum* L. cv. Skyfall) and an AM Fungus (*Rhizophagus irregularis*)

Experimental systems were established at ambient (aCO_2; 440 ppm) and elevated (eCO_2; 800 ppm) atmospheric [CO_2], and plants were either exposed to the bird cherry-oat aphid (*Rhopalosiphum padi*) or not during the labeling period.

(A) ^{33}P-labeled orthophosphate was introduced to mesh-walled cores accessible only to fungal mycelia of the AM fungus. Mycorrhiza-acquired ^{33}P was calculated by subtracting quantities of isotope tracer recorded in shoots of plants with "rotated" labeled cores (shown) from those in which labeled cores were kept "static."

(B) Pots were sealed within airtight chambers, and $^{14}CO_2$ was liberated from ^{14}C-labeled sodium bicarbonate into the headspace of plants. $^{14}CO_2$ was fixed by plants and allocated to the extra-radical mycelium of the AM fungus or assimilated by aphids within insect clip cages fixed to the third leaf on the main tiller of each plant.

See also Figure S1 and STAR Methods.

strength of plants by inducing defense-signaling pathways [25] and/or altering rates of photosynthesis [26]. As such, although highly variable, aphid infestation can cause reduced colonization of plant roots by AM fungi [27, 28], potentially as a result of declining plant C availability for mycorrhizal symbionts [23]. This "C-limitation" mechanism following herbivory has been hypothesized across plant functional groups [29] and could compromise transfer of fungal-acquired nutrients to host plants if resource exchange between symbionts is regulated symmetrically [18]. However, the intensity of AM colonization within a plant root system is often a poor predictor of mycorrhizal function [30, 31]. As such, using this metric alone to infer changes in symbiotic function after aphid infestation could be misleading.

We investigated the effect of manipulating the source and sink strengths of plant C resources on carbon for nutrient exchange between wheat and a cooperative [18], widely distributed AM fungus (*Rhizophagus irregularis*) [32], which both have economic, ecological, and societal relevance. C source strength, and thus availability of plant C for the AM fungus, was increased by changing atmospheric [CO_2] in line with future climate predictions [33]. C sink strength was manipulated by the addition or exclusion of bird cherry-oat aphids (*Rhopalosiphum padi*), which served to either increase or reduce competition for (and thus availability of) plant C resources to AM. In this ecologically relevant tri-trophic system (Figure 1), we addressed the following questions:

(1) does increasing external C sink strength (i.e., addition of aphids) reduce recently fixed plant C allocation to an AM fungus?
(2) does increasing C source strength (i.e., elevated [CO_2]) increase recently fixed plant C allocation to an AM fungus?
(3) can increasing C source strength mitigate increased external plant C sinks?
(4) does plant assimilation of mycorrhizal-acquired P change relative to recently fixed plant C allocation?

Increasing external C sink strength through aphid exposure might be expected to reduce the availability, and thus allocation, of plant C to the AM fungus [23]. If plant nutrient gain via the AM fungus is directly linked to plant C allocation [18], the amount of fungal-acquired P transferred to the plant would be reduced when external C sink strength is increased. In contrast, elevated [CO_2], which may increase plant C source strength for AM fungi [13], is expected to mitigate the effects of an aphid-induced increased C sink and restore mycorrhizal-acquired nutrient transfer to plant hosts.

RESULTS

[CO_2] and Aphid Herbivory Modify Host-Plant C Availability

In order to manipulate the source and sink strength of wheat C resources, plants were grown at ambient (aCO_2; 440 ppm) and elevated (eCO_2; 800 ppm) [CO_2] and exposed or not exposed to a specialist phloem feeding herbivore of cereals, the bird cherry-oat aphid (*R. padi*; see STAR Methods).

Wheat plants grown at eCO_2 were larger above ground than plants grown at aCO_2 (Figure 2A; $F_{1,44} = 52.19$; $p < 0.001$), as with previous studies [34], regardless of whether plants were exposed or not to aphid herbivores (− aphids: +28%; + aphids: +30%). eCO_2 also increased shoot C concentrations (Figure S2A; Table S1), suggesting that host plants grown at eCO_2 represented greater C source strengths than those at aCO_2. Aphid herbivory reduced shoot biomass at aCO_2 and eCO_2 by 14% and 11%, respectively ($F_{1,44} = 16.01$; $p < 0.001$).

There was no effect of [CO_2] on below-ground wheat biomass (Figure 2B; $F_{1,44} = 0.01$; $p = 0.931$); however, root C concentrations were greater at eCO_2 compared to aCO_2 (Figure S2B; Table S1). Aphids dramatically reduced root biomass ($F_{1,44} = 172.48$; $p < 0.001$) under both [CO_2] treatments (aCO_2: −54%; eCO_2: −57%), in agreement with prior work on aphid-infested spring

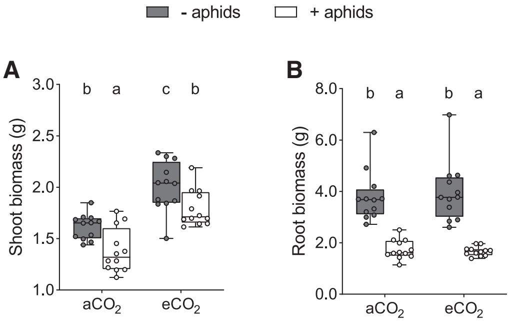

Figure 2. Biomass of Plants Not Exposed (Gray Boxes) or Exposed (White Boxes) to Aphids at Ambient and Elevated Atmospheric [CO_2]

(A) Shoot biomass (dry weight).

(B) Root biomass (dry weight). Boxplots extend from the first to the third quartile, with the middle line representing median values (n = 12). Whiskers are drawn to the minimum and maximum data points (open or closed markers). Different letters denote significant differences between treatment means (where $p < 0.05$, generalized linear model [GLM] and Tukey honest significant difference [HSD] tests).

See also Figures S2A and S2B.

wheat [35] and Timothy grass (*Phleum pratense*) [36]. There was no interactive effect of [CO_2] and aphids on shoot ($F_{1,44} = 0.02$; $p = 0.885$) or root biomass ($F_{1,44} = 0.23$; $p = 0.636$), as despite aphid population growth rates being greater at eCO_2 than at aCO_2 (Figure S3A), final aphid abundance (Figure S3B) and the amount of recently fixed plant C assimilated by aphids (i.e., external biotic C sink strengths) were the same across [CO_2] treatments (Figures S3C and S3D).

AM Fungal Responses to [CO_2] and Aphid Herbivory

Next, we assessed the effect of increasing C source and sink strengths on root-internal and root-external abundances of the AM fungus. Staining of wheat roots with acidified ink (see STAR Methods) confirmed that all plants were colonized by the arbuscular mycorrhizal fungus. Counter to previous findings [14], % root length colonization by the AM fungus was lower in plants grown at eCO_2 compared to those under aCO_2 (Figure 3A; $F_{1,44} = 14.94$; $p < 0.001$) in both aphid treatments (− aphids: −44%; + aphids: −29%). In contrast, exposure to aphids resulted in greater % AM fungal colonization of plant roots (aCO_2: +41%; eCO_2: +79%; $F_{1,44} = 14.73$; $p < 0.001$), although these root systems were considerably smaller. No interaction between [CO_2] and aphid herbivory was recorded on % root length colonization by the AM fungus ($F_{1,44} = 0.05$; $p = 0.823$). Trends were consistent for arbuscule and vesicle frequencies within wheat roots (Figures S2C and S2D; Table S1), these being fungal structures thought to be involved principally in resource exchange and storage, respectively [8].

Extra-radical fungal hyphae were extracted from bulk substrates of plants, and root-external mycorrhizal abundances were quantified (see STAR Methods). There was no effect of [CO_2] ($F_{1,44} = 0.06$; $p = 0.810$) or aphids ($F_{1,44} = 0.34$; $p = 0.565$) on the length of AM fungal hyphae supported by wheat roots (Figure 3B).

Aphid Herbivory Reduces Plant C Allocation to an AM Fungus

Changes in % root length colonization by the AM fungus at high [CO_2] and following aphid exposure could suggest modified plant C allocation to the fungal symbiont. However, it has been shown that AM presence or abundance in roots of plants may not reliably correlate with physiological function in plant-AM symbioses [30, 31]. As such, in order to directly test the effect of C sink-source strength dynamics on plant C allocation to the AM fungus, wheat was supplied with a ^{14}C-labeled pulse of CO_2 within an airtight chamber and the allocation of recently fixed plant C to the root mutualist was quantified (Figure 1B; see STAR Methods).

Transfer of plant C to the AM fungus was dramatically reduced in plants exposed to aphids compared to those that were not (Figure 4A; Table S2) by 97% and 73% at aCO_2 and eCO_2, respectively. This finding was in line with the C-limitation hypothesis [23, 29]. In contrast, wheat grown at eCO_2 transferred similar amounts of recently fixed C to the AM fungus as plants grown in aCO_2 (Table S2), at odds with previous studies that suggest AM fungi receive greater plant C allocation when C source strengths increase [13]. When expressed as a % of plant-fixed C, aphid exposure similarly reduced plant C distribution to the AM fungus (Figure 4B; $F_{1,44} = 47.89$; $p < 0.001$), but atmospheric [CO_2] had no effect ($F_{1,44} = 2.96$; $p = 0.092$).

AM-Acquired ^{33}P Uptake Was Not Linked to Plant C Allocation

Lastly, we assessed the effect of C sink-source strength dynamics on plant- and AM fungal-acquired phosphorous (P) uptake (see STAR Methods). Total shoot P concentration, this being plant- and mycorrhizal-mediated, was significantly lower in wheat grown under eCO_2 compared to aCO_2 (Figure 5A; $F_{1,44} = 16.77$; $p < 0.001$) and in plants exposed to aphids compared to those that were not ($F_{1,44} = 63.98$; $p < 0.001$). No interaction between [CO_2] and aphids was recorded ($F_{1,44} = 2.93$; $p = 0.094$). In order to quantify how plant C provisioning impacted plant P assimilation via the AM fungus alone, ^{33}P-labeled orthophosphate was introduced to regions of substrate accessible only to fungal hyphae of the AM fungus and its assimilation into the plant quantified through liquid scintillation (Figure 1A; see STAR Methods).

We determined that, at eCO_2, fungal-mediated shoot ^{33}P concentration ([^{33}P]) was greater in plants exposed to aphids than those that were not (Figure 5B; $F_{1,20} = 4.36$; $p = 0.049$). This was despite plant C allocation to the AM fungus being reduced following increased C sink strength (Figure 4), suggesting aphids drove asymmetry in carbon for nutrient exchange between mycorrhizal symbionts. Shoot [^{33}P] was also greater in aphid-exposed plants at eCO_2 than in plants grown at aCO_2 ($F_{1,20} = 4.36$; $p = 0.049$), with between 3% and 11% of the ^{33}P tracer supplied to the AM fungus recovered in plant shoot tissues across treatments. Similar patterns were recorded for total shoot P and ^{33}P (Figures 5C and 5D). No correlation was recorded between % root length colonization by the AM fungus and mycorrhizal-acquired shoot ^{33}P (Figures S4A and S4B) or between plant C outlay and shoot ^{33}P (Figures S4C and S4D), suggesting mycorrhizal function was not related to fungal abundance within the roots [30, 31] or recently fixed plant C allocation.

Root P (Figure S2E; Table S1) was lower in aphid-exposed plants, although, in contrast, root [P], root ^{33}P, and root [^{33}P] (Figures S2F–S2H; Table S1) were greater in plants exposed to aphids. However, root P and ^{33}P values inevitably include

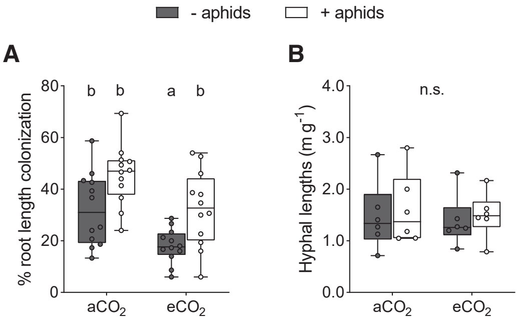

Figure 3. AM Fungal Colonization of Roots and the Extent of AM Fungal Hyphal Network in Substrates of Plants Not Exposed (Gray Boxes) or Exposed (White Boxes) to Aphids at Ambient and Elevated Atmospheric [CO_2]

(A) % root length colonization.

(B) Extra-radical hyphal lengths in surrounding substrate. Boxplots extend from the first to the third quartile, with the middle line representing median values (n = 12 for A; n = 6 for B). Whiskers are drawn to the minimum and maximum data points (open or closed markers). Different letters denote significant differences between treatment means (where $p < 0.05$, GLM + Tukey HSD tests). "n.s." denotes no significant difference between treatment means. See also Figures S2C, S2D, and S4.

phosphorous held within fungal structures in the root cortex, such as intracellular hyphae and arbuscules. Thus, root values do not permit us to make inferences about plant assimilation of AM-fungal-acquired P. ^{33}P was also recorded in the AM fungal hyphal network at harvest, perhaps being translocated toward the root, with values similar to those of ^{33}P concentrations in the shoot (Figure S2I; Table S1).

DISCUSSION

Carbon for nutrient exchange between AM fungi and their host plants is widely considered characteristic of arbuscular mycorrhizal symbioses and has sparked interest in recent years in the potential exploitation of AM fungi for agronomic gain [37]. However, in nature, plants seldom interact with AM fungi in isolation. Instead, it is common for plants to simultaneously interact with a variety of other organisms within a dynamic environment [38]. To date, the impact of simultaneous, interacting abiotic and biotic factors on resource exchange between plants and AM fungi has not been tested. We examined how manipulating C source and sink strengths in an ecologically relevant, tri-partite system impacted plant C allocation to an AM fungus and AM-fungal-mediated plant P assimilation.

Increasing C sink strength through the addition of aphids almost eliminated recently fixed plant C allocation to the AM fungus (Figure 4) although, despite this dramatic effect, the transfer of AM-fungal-acquired ^{33}P to host plants was maintained (Figures 5B and 5D). Increasing C source strength by growing plants in a high-[CO_2] atmosphere failed to restore plant C allocation to the AM fungus but resulted in increased transfer of ^{33}P from AM fungi to host plants, potentially as a result of increased demand for plant resources driven by a growing aphid population (Figure S3A). Our findings highlight the context dependency of carbon for nutrient exchanges between plants and AM fungi in complex biological systems.

Increasing C Sink Strength Reduces Allocation of Plant C to an AM Fungus

Allocation of recently fixed plant C to the AM fungus in our experiment was dramatically reduced when plants were exposed to aphids and did not increase at eCO_2 (Figure 4). This finding supports the C-limitation hypothesis [23]; aphids reduced plant C availability for the AM fungus by directly siphoning plant C via phloem feeding (Figures S3C and S3D) and may have further limited plant C resources by inducing defense-signaling pathways [7, 25] and/or the production of carbohydrate-rich secondary metabolites [39]. The dramatic reduction in plant C allocation to the AM fungus was the same across [CO_2] treatments, despite differences in *R. padi* growth rates (Figure S3A). Although, to the best of our knowledge, the effect of aphids on plant C allocation to the extra-radical mycelium of an AM fungus has not been quantified before, our findings confirm the strong impact of phloem-feeding herbivores on the C budget of target plants [26], with previous studies recording systemic changes in plant C partitioning following short-term aphid exposure [40].

As obligate biotrophs [8], AM fungi rely exclusively on their plant host for C resources. Intracellular plant-fungal interfaces form and degenerate throughout the lifetime of the symbiosis [41]. As such, the degree to which roots were colonized by AM fungi and their associated extra-radical hyphal networks was determined using cytological methods. This method may be used to infer relative plant C investment over longer time periods than the instantaneous measurements made using isotope-tracing approaches [42]. However, when considered alone, the intensity of AM fungal colonization within a plant root system does not always reflect mycorrhizal function [30, 31]. Taking this caveat into account, when plant C becomes limited, or external C sink strengths increase, root colonization might be expected to decline [23]. However, in our experiment, AM fungal colonization was greater in plants that were exposed to aphids than those that were not, under both CO_2 atmospheres (Figure 3A). Using the same cytological methods, negative, neutral, and positive effects of aphid herbivory on AM colonization have been recorded across plant-AM-aphid systems [27, 43], with idiosyncratic outcomes even documented between plant species within the same genus [28]. The increase in % root length colonization recorded here may have been driven by a reduction in root biomass of plants exposure to aphid herbivores (Figure 2B), with total fungal presence potentially being unchanged in roots between aphid treatments. Declining root biomass of wheat following aphid infestation has been observed previously in spring wheat [35] and a perennial grass species [36], as well as in other plant-aphid combinations [44], emphasizing that root colonization by AM fungi can be a poor indicator of symbiotic function, particularly within multi-trophic contexts. Quantification of metabolically active AM fungal abundance using a qPCR approach could have been beneficial in this instance, but evidence suggests such approaches for assessing AM colonization are, similarly, not definitive [45, 46].

Plants grown at eCO_2 were larger and had greater shoot and root C concentrations than those grown in aCO_2 (Figures 2A, S2A, and S2B), suggesting that more plant-fixed C was available for the AM fungus under eCO_2 conditions, with the potential to mitigate the loss of plant C via aphid herbivory. However, there was no change in root biomass (Figure 2B) or recently fixed plant

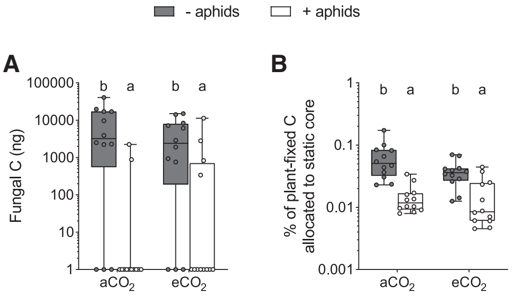

Figure 4. Plant Carbon (C) Allocation to the AM Fungus when Not Exposed (Gray Boxes) or Exposed (White Boxes) to Aphids at Ambient and Elevated Atmospheric [CO_2]

(A) Transfer of recently fixed plant C to the AM hyphal network in the pot (log scale).

(B) % of recently fixed plant C recovered in the static core (log scale). Boxplots extend from the first to the third quartile, with the middle line representing median values (n = 12). Whiskers are drawn to the minimum and maximum data points (open or closed markers). Different letters denote significant differences between treatment means (where $p < 0.05$, GLM + Tukey HSD tests, except for A, which were determined using multiple Mann-Whitney U tests; see Table S2). The effect of [CO_2] on aphids is displayed in Figure S3. AM transfer of ^{33}P in relation to plant C allocation is displayed in Figure S4.

C allocation to the AM fungus at eCO_2 (Figure 4), contrasting with previous findings in wild plants [13]. The amount of recently fixed plant C allocated to the AM fungus in our experiment was lower than that reported for other plant species [10, 47], likely reflecting the low mycorrhizal receptivity and function of wheat [48, 49]. Selective breeding for above-ground, yield-related characteristics, such as disease resistance and responsiveness to high nutrient inputs in modern cereal cultivars, may have inadvertently selected against below-ground traits, such as root growth [50] and AM fungal receptivity [37]. Consequently, modern cultivars typically have lower root-to-shoot ratios than older varieties [51] and may allocate less plant C to fungal symbionts than wild plants, even in favorable conditions where plant-fixed C resources are readily available [48]. We recorded lower % root length colonization by the AM fungus at eCO_2 compared to aCO_2 (Figure 3A), suggesting that longer term plant C allocation to the AM fungus may have even been lower at eCO_2 than at aCO_2 over the entire plant growth period. Together, these results demonstrate that increased availability of plant C does not always result in greater C allocation to AM fungi and may not mitigate plant C losses to insect herbivores. Future studies involving plant hosts that vary in below-ground allocation of resources and mycorrhizal receptivity are now required to determine whether biotic and abiotic factors that affect sink-source dynamics impact recently fixed plant C allocation to AM fungi similarly across plant functional groups.

AM-Fungal-Acquired P Is Not Related to Plant C Allocation

In most cases, plants assimilate P directly via their roots instead of, or in addition to, via mycorrhizal fungi [52]. As a result, plant P assimilation typically represents the sum of P uptake by these two pathways, with AM fungi rarely being entirely responsible for plant P acquisition. In our experiment, total above-ground plant [P] declined when C sink strength increased following aphid exposure and to a greater extent at eCO_2 when more aphids were present (Figure 5A). By using a ^{33}P tracer, we determined that the amount of AM-acquired ^{33}P assimilated into plant tissues was unaffected by aphids at aCO_2, and increased in their presence at eCO_2 (Figures 5B and 5D). Together with our finding that aphids caused a dramatic reduction in plant C allocation to the AM fungus, our data suggest that plant C allocation to AM fungi was asymmetrically linked to mycorrhizal-acquired P in the presence of aphids at the time of sampling. Instead, lower shoot [P] and P (Figures 5A and 5C) was likely a consequence of reduced root biomass in aphid-exposed plants, thereby impairing the effectiveness of root foraging and the plant P assimilation pathway.

Asymmetry in carbon for nutrient exchange between the AM fungus and host plant is further evidenced by AM-acquired ^{33}P being greater at eCO_2, despite there being equivalent plant C allocation to the AM fungus and faster aphid population growth. According to our earlier hypothesis, increasing availability of [CO_2] for photosynthesis was predicted to increase plant C source strength for AM fungi and in turn increase movement of AM-fungal-acquired P to the host plant [18]. However, our results do not provide evidence for this, as plant C provisioning of the AM fungus was unaffected by [CO_2]. Instead, allocation of recently fixed plant C to the AM fungus was dramatically affected by an external biotic C sink (Figure 4), confirming the context dependency of carbon for nutrient exchange. Intriguingly, mycorrhizal P acquisition was not determined by the degree to which plant root systems were colonized by the AM fungus (Figures S4A and S4B), as recorded previously in maize [31], or linked to recently fixed plant C allocation to the fungus (Figures S4C and S4D).

Herbivore-induced asymmetry in carbon for nutrient exchange between mycorrhizal symbionts could suggest that resource exchange is not coordinated reciprocally [18, 19] in ecologically relevant, tri-partite systems. An alternative explanation for this apparent breakdown in symmetrically regulated resource exchange may be the lack of another host for the AM fungus—and therefore C source—in our experiment. This may have prohibited *R. irregularis* from "sanctioning" its plant partner by reducing ^{33}P assimilation, as to do so may have reduced plant tolerance to herbivory [53] and/or limited subsequent plant C allocation to the AM fungus. Future studies must now seek to investigate the effect of external biotic C sinks on resource exchange between AM fungi and multiple host plants (i.e., multiple C sources) in more complex, and ecologically relevant, networks.

Lifetime fitness benefits of AM symbioses to plant hosts are likely important in regulating resource transfer between partners, with non-nutritional benefits of the symbiosis [5, 6] also playing a significant role in regulating resource exchange. Our isotope-tracing approach was limited to measurement of plant C for fungal P over a relatively short time period. As such, our results do not account for AM fungal contributions to plant P or for plant C allocation to the AM fungus outside of the isotope labeling window or the wider impacts of aphid herbivory on plant-AM functionality across the life cycle of the plant. Our experiment was conducted during the shoot elongation growth stage of wheat [54], enabling assessment of sink-source dynamics on carbon for nutrient exchange between mycorrhizal symbionts during a

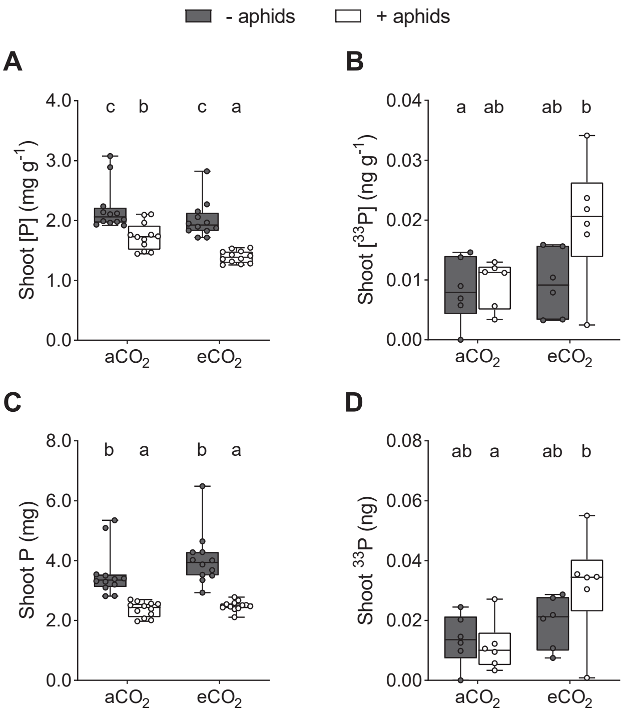

Figure 5. Phosphorous (P) Uptake by Plants and the AM Fungus when Not Exposed (Gray Boxes) and Exposed (White Boxes) to Aphid Herbivores at Ambient and Elevated Atmospheric [CO_2]

(A) Shoot P concentration.

(B) AM fungal-acquired shoot ^{33}P concentration.

(C) Shoot P content.

(D) AM fungal-acquired shoot ^{33}P content. Boxplots extend from the first to the third quartile, with the middle line representing median values (n = 12, except for B and D, where n = 6). Whiskers are drawn to the minimum and maximum data points (open or closed markers). Different letters denote significant differences between treatment means (where $p < 0.05$, GLM + Tukey HSD tests). Root P, [P], ^{33}P, and [^{33}P] are displayed in Figure S2. AM transfer of ^{33}P in relation to % root length colonization and plant C allocation is displayed in Figure S4. Calibration curve for determination of P is displayed in Figure S5.

critical growth stage of high nutrient demand [55]. Future studies should look to investigate the impact of biotic and abiotic factors on resource exchange across multiple time points, given the functionality of AM symbioses in wheat is likely to shift during different growth phases [56], for instance, when plant resources are remobilized from roots and shoots to ears during grain filling [57]. Nonetheless, our results provide an important insight into how biotic and abiotic factors influence resource exchange between mycorrhizal symbionts and how plant-AM-herbivore interactions could be influenced by predicted future increases in [CO_2] [38]. Further research is now needed to monitor resource exchange between symbionts across plant life histories and in more complex environments involving multiple host plants and fungal diversity, while accounting for the simultaneous influence of both abiotic and other biotic drivers.

STAR★METHODS

Detailed methods are provided in the online version of this paper and include the following:

- KEY RESOURCES TABLE
- LEAD CONTACT AND MATERIALS AVAILABILITY
- EXPERIMENTAL MODEL AND SUBJECT DETAILS
  - Plant material and growth conditions
  - Fungal material and culture conditions
  - Aphid material and culture conditions
- METHOD DETAILS
  - Experimental set-up
  - Aphid exposure
  - ^{33}P isotope tracing
  - ^{14}C label
  - Plant harvest and sample preparation
  - AM fungal colonization of roots and bulk substrates
  - Plant- and mycorrhizal-acquired P and ^{33}P
  - Plant C transfer to the AM fungus and assimilation by aphids
- QUANTIFICATION AND STATISTICAL ANALYSIS
  - Data analyses
- DATA AND CODE AVAILABILITY

SUPPLEMENTAL INFORMATION

Supplemental Information can be found online at https://doi.org/10.1016/j.cub.2020.02.087.

ACKNOWLEDGMENTS

We thank Tom Pope (Harper Adams University) for providing aphids used to establish our own cultures and Thomas Thirkell, Grace Hoysted, Ashleigh Elliott, Bev Merry, and Daria Pastok for their assistance with plant harvest. Funding was provided from the Biotechnology and Biological Sciences Research Council BB/M026825/1 (to K.J.F.), Rank Prize Funds New Lecturer Award (to K.J.F.), and the Leeds-York Natural Environment Research Council Doctoral Training Partnership "Spheres" (to M.D.C.). We thank the editor and three anonymous reviewers for their much-valued constructive comments during peer review.

AUTHOR CONTRIBUTIONS

M.D.C., S.M.S., and K.J.F. conceived and designed the investigation. M.D.C. conducted experiments, analyzed the data, and wrote the first draft of the manuscript with assistance from S.M.S. and K.J.F. All authors discussed results and commented on the manuscript. K.J.F. agrees to serve as the author responsible for correspondence.

DECLARATION OF INTERESTS

The authors declare no competing interests.

Received: December 3, 2019
Revised: January 28, 2020
Accepted: February 27, 2020
Published: April 9, 2020

REFERENCES

1. Brundrett, M.C., and Tedersoo, L. (2018). Evolutionary history of mycorrhizal symbioses and global host plant diversity. New Phytol. *220*, 1108–1115.
2. Field, K.J., and Pressel, S. (2018). Unity in diversity: structural and functional insights into the ancient partnerships between plants and fungi. New Phytol. *220*, 996–1011.
3. Pirozynski, K.A., and Malloch, D.W. (1975). The origin of land plants: a matter of mycotrophism. Biosystems *6*, 153–164.

4. Helgason, T., and Fitter, A.H. (2009). Natural selection and the evolutionary ecology of the arbuscular mycorrhizal fungi (Phylum Glomeromycota). J. Exp. Bot. *60*, 2465–2480.
5. Sikes, B.A., Cottenie, K., and Klironomos, J.N. (2009). Plant and fungal identity determines pathogen protection of plant roots by arbuscular mycorrhizas. J. Ecol. *97*, 1274–1280.
6. Koricheva, J., Gange, A.C., and Jones, T. (2009). Effects of mycorrhizal fungi on insect herbivores: a meta-analysis. Ecology *90*, 2088–2097.
7. Cameron, D.D., Neal, A.L., van Wees, S.C.M., and Ton, J. (2013). Mycorrhiza-induced resistance: more than the sum of its parts? Trends Plant Sci. *18*, 539–545.
8. Smith, S.E., and Read, D.J. (2010). Mycorrhizal Symbiosis (Academic).
9. Roth, R., and Paszkowski, U. (2017). Plant carbon nourishment of arbuscular mycorrhizal fungi. Curr. Opin. Plant Biol. *39*, 50–56.
10. Field, K.J., Davidson, S.J., Alghamdi, S.A., and Cameron, D.D. (2017). Magnitude, dynamics, and control of the carbon flow to mycorrhizas. In Mycorrhizal Mediation of Soil, N.C. Johnson, C. Gehring, and J. Jansa, eds. (Elsevier), pp. 375–393.
11. Luginbuehl, L.H., Menard, G.N., Kurup, S., Van Erp, H., Radhakrishnan, G.V., Breakspear, A., Oldroyd, G.E.D., and Eastmond, P.J. (2017). Fatty acids in arbuscular mycorrhizal fungi are synthesized by the host plant. Science *356*, 1175–1178.
12. Lendenmann, M., Thonar, C., Barnard, R.L., Salmon, Y., Werner, R.A., Frossard, E., and Jansa, J. (2011). Symbiont identity matters: carbon and phosphorus fluxes between *Medicago truncatula* and different arbuscular mycorrhizal fungi. Mycorrhiza *21*, 689–702.
13. Drigo, B., Pijl, A.S., Duyts, H., Kielak, A.M., Gamper, H.A., Houtekamer, M.J., Boschker, H.T.S., Bodelier, P.L.E., Whiteley, A.S., van Veen, J.A., and Kowalchuk, G.A. (2010). Shifting carbon flow from roots into associated microbial communities in response to elevated atmospheric CO_2. Proc. Natl. Acad. Sci. USA *107*, 10938–10942.
14. Dong, Y., Wang, Z., Sun, H., Yang, W., and Xu, H. (2018). The response patterns of arbuscular mycorrhizal and ectomycorrhizal symbionts under elevated CO_2: a meta-analysis. Front. Microbiol. *9*, 1248.
15. Ainsworth, E.A., and Rogers, A. (2007). The response of photosynthesis and stomatal conductance to rising [CO_2]: mechanisms and environmental interactions. Plant Cell Environ. *30*, 258–270.
16. Hammer, E.C., Pallon, J., Wallander, H., and Olsson, P.A. (2011). Tit for tat? A mycorrhizal fungus accumulates phosphorus under low plant carbon availability. FEMS Microbiol. Ecol. *76*, 236–244.
17. Bever, J.D., Richardson, S.C., Lawrence, B.M., Holmes, J., and Watson, M. (2009). Preferential allocation to beneficial symbiont with spatial structure maintains mycorrhizal mutualism. Ecol. Lett. *12*, 13–21.
18. Kiers, E.T., Duhamel, M., Beesetty, Y., Mensah, J.A., Franken, O., Verbruggen, E., Fellbaum, C.R., Kowalchuk, G.A., Hart, M.M., Bago, A., et al. (2011). Reciprocal rewards stabilize cooperation in the mycorrhizal symbiosis. Science *333*, 880–882.
19. Fellbaum, C.R., Gachomo, E.W., Beesetty, Y., Choudhari, S., Strahan, G.D., Pfeffer, P.E., Kiers, E.T., and Bücking, H. (2012). Carbon availability triggers fungal nitrogen uptake and transport in arbuscular mycorrhizal symbiosis. Proc. Natl. Acad. Sci. USA *109*, 2666–2671.
20. Walder, F., and van der Heijden, M.G.A. (2015). Regulation of resource exchange in the arbuscular mycorrhizal symbiosis. Nat. Plants *1*, 15159.
21. Walder, F., Niemann, H., Natarajan, M., Lehmann, M.F., Boller, T., and Wiemken, A. (2012). Mycorrhizal networks: common goods of plants shared under unequal terms of trade. Plant Physiol. *159*, 789–797.
22. Field, K.J., Cameron, D.D., Leake, J.R., Tille, S., Bidartondo, M.I., and Beerling, D.J. (2012). Contrasting arbuscular mycorrhizal responses of vascular and non-vascular plants to a simulated Palaeozoic CO_2 decline. Nat. Commun. *3*, 835.
23. Gehring, C.A., and Whitham, T.G. (2002). Mycorrhizae-herbivore interactions: population and community consequences. In Mycorrhizal Ecology, M.G.A. van der Heijden, and I.R. Sanders, eds. (Springer), pp. 295–320.
24. Donovan, M.P., Nabity, P.D., and DeLucia, E.H. (2013). Salicylic acid-mediated reductions in yield in *Nicotiana attenuata* challenged by aphid herbivory. Arthropod-Plant Interact. *7*, 42–52.
25. Ali, J.G., and Agrawal, A.A. (2012). Specialist versus generalist insect herbivores and plant defense. Trends Plant Sci. *17*, 293–302.
26. Macedo, T.B., Peterson, R.K.D., Weaver, D.K., and Ni, X. (2009). Impact of *Diuraphis noxia* and *Rhopalosiphum padi* (Hemiptera: Aphididae) on primary physiology of four near-isogenic wheat lines. J. Econ. Entomol. *102*, 412–421.
27. Babikova, Z., Gilbert, L., Bruce, T., Dewhirst, S.Y., Pickett, J.A., and Johnson, D. (2014). Arbuscular mycorrhizal fungi and aphids interact by changing host plant quality and volatile emission. Funct. Ecol. *28*, 375–385.
28. Meier, A.R., and Hunter, M.D. (2018). Arbuscular mycorrhizal fungi mediate herbivore-induction of plant defenses differently above and belowground. Oikos *127*, 1759–1775.
29. Barto, E.K., and Rillig, M.C. (2010). Does herbivory really suppress mycorrhiza? A meta-analysis. J. Ecol. *98*, 745–753.
30. Nagy, R., Drissner, D., Amrhein, N., Jakobsen, I., and Bucher, M. (2009). Mycorrhizal phosphate uptake pathway in tomato is phosphorus-repressible and transcriptionally regulated. New Phytol. *181*, 950–959.
31. Sawers, R.J.H., Svane, S.F., Quan, C., Grønlund, M., Wozniak, B., Gebreselassie, M.-N., González-Muñoz, E., Chávez Montes, R.A., Baxter, I., Goudet, J., et al. (2017). Phosphorus acquisition efficiency in arbuscular mycorrhizal maize is correlated with the abundance of root-external hyphae and the accumulation of transcripts encoding PHT1 phosphate transporters. New Phytol. *214*, 632–643.
32. Savary, R., Masclaux, F.G., Wyss, T., Droh, G., Cruz Corella, J., Machado, A.P., Morton, J.B., and Sanders, I.R. (2018). A population genomics approach shows widespread geographical distribution of cryptic genomic forms of the symbiotic fungus *Rhizophagus irregularis*. ISME J. *12*, 17–30.
33. Meinshausen, M., Smith, S.J., Calvin, K., Daniel, J.S., Kainuma, M.L.T., Lamarque, J.-F., Matsumoto, K., Montzka, S.A., Raper, S.C.B., Riahi, K., et al. (2011). The RCP greenhouse gas concentrations and their extensions from 1765 to 2300. Clim. Change *109*, 213.
34. Amthor, J.S. (2001). Effects of atmospheric CO_2 concentration on wheat yield: review of results from experiments using various approaches to control CO_2 concentration. Field Crops Res. *73*, 1–34.
35. Riedell, W.E., and Kieckhefer, R.W. (1995). Feeding damage effects of three aphid species on wheat root growth. J. Plant Nutr. *18*, 1881–1891.
36. Hempel, S., Stein, C., Unsicker, S.B., Renker, C., Auge, H., Weisser, W.W., and Buscot, F. (2009). Specific bottom-up effects of arbuscular mycorrhizal fungi across a plant-herbivore-parasitoid system. Oecologia *160*, 267–277.
37. Thirkell, T.J., Charters, M.D., Elliott, A.J., Sait, S.M., and Field, K.J. (2017). Are mycorrhizal fungi our sustainable saviours? Considerations for achieving food security. J. Ecol. *105*, 921–929.
38. Frew, A., and Price, J.N. (2019). Mycorrhizal-mediated plant-herbivore interactions in a high CO_2 world. Funct. Ecol. *33*, 1376–1385.
39. Ahmad, S., Veyrat, N., Gordon-Weeks, R., Zhang, Y., Martin, J., Smart, L., Glauser, G., Erb, M., Flors, V., Frey, M., and Ton, J. (2011). Benzoxazinoid metabolites regulate innate immunity against aphids and fungi in maize. Plant Physiol. *157*, 317–327.
40. Girousse, C., Faucher, M., Kleinpeter, C., and Bonnemain, J.-L. (2003). Dissection of the effects of the aphid *Acyrthosiphon pisum* feeding on assimilate partitioning in *Medicago sativa*. New Phytol. *157*, 83–92.
41. Luginbuehl, L.H., and Oldroyd, G.E.D. (2017). Understanding the arbuscule at the heart of endomycorrhizal symbioses in plants. Curr. Biol. *27*, R952–R963.
42. Müller, A., Ngwene, B., Peiter, E., and George, E. (2017). Quantity and distribution of arbuscular mycorrhizal fungal storage organs within dead roots. Mycorrhiza *27*, 201–210.

43. Wilkinson, T.D.J., Miranda, J.-P., Ferrari, J., Hartley, S.E., and Hodge, A. (2019). Aphids influence soil fungal communities in conventional agricultural systems. Front. Plant Sci. *10*, 895.
44. Hoysted, G.A., Bell, C.A., Lilley, C.J., and Urwin, P.E. (2018). Aphid colonization affects potato root exudate composition and the hatching of a soil borne pathogen. Front. Plant Sci. *9*, 1278.
45. Thonar, C., Erb, A., and Jansa, J. (2012). Real-time PCR to quantify composition of arbuscular mycorrhizal fungal communities–marker design, verification, calibration and field validation. Mol. Ecol. Resour. *12*, 219–232.
46. Voříšková, A., Jansa, J., Püschel, D., Krüger, M., Cajthaml, T., Vosátka, M., and Janoušková, M. (2017). Real-time PCR quantification of arbuscular mycorrhizal fungi: does the use of nuclear or mitochondrial markers make a difference? Mycorrhiza *27*, 577–585.
47. Tomè, E., Tagliavini, M., and Scandellari, F. (2015). Recently fixed carbon allocation in strawberry plants and concurrent inorganic nitrogen uptake through arbuscular mycorrhizal fungi. J. Plant Physiol. *179*, 83–89.
48. Thirkell, T.J., Pastok, D., and Field, K.J. (2020). Carbon for nutrient exchange between arbuscular mycorrhizal fungi and wheat varies according to cultivar and changes in atmospheric carbon dioxide concentration. Glob. Change Biol. *26*, 1725–1738.
49. Elliott, A.J., Daniell, T.J., Cameron, D.D., and Field, K.J. (2020). A commercial arbuscular mycorrhizal inoculum increases root colonization across wheat cultivars but does not increase assimilation of mycorrhizal-acquired nutrients. Plants People Planet. Published online January 3, 2020. https://doi.org/10.1002/ppp3.10094.
50. Voss-Fels, K.P., Qian, L., Parra-Londono, S., Uptmoor, R., Frisch, M., Keeble-Gagnère, G., Appels, R., and Snowdon, R.J. (2017). Linkage drag constrains the roots of modern wheat. Plant Cell Environ. *40*, 717–725.
51. Siddique, K.H.M., Belford, R.K., and Tennant, D. (1990). Root:shoot ratios of old and modern, tall and semi-dwarf wheats in a mediterranean environment. Plant Soil *121*, 89–98.
52. Smith, S.E., and Smith, F.A. (2011). Roles of arbuscular mycorrhizas in plant nutrition and growth: new paradigms from cellular to ecosystem scales. Annu. Rev. Plant Biol. *62*, 227–250.
53. Maschinski, J., and Whitham, T.G. (1989). The continuum of plant responses to herbivory: the influence of plant association, nutrient availability, and timing. Am. Nat. *134*, 1–19.
54. Zadocks, J.C., Chang, T.T., and Konzak, C.F. (1974). A decimal code for the growth stages of cereals. Weed Res. *14*, 415–421.
55. Weih, M., Pourazari, F., and Vico, G. (2016). Nutrient stoichiometry in winter wheat: Element concentration pattern reflects developmental stage and weather. Sci. Rep. *6*, 35958.
56. Johnson, N.C., Graham, J.-H., and Smith, F.A. (1997). Functioning of mycorrhizal associations along the mutualism–parasitism continuum. New Phytol. *135*, 575–585.
57. Shewry, P.R. (2009). Wheat. J. Exp. Bot. *60*, 1537–1553.
58. RAGT (2018). RGT Skyfall G1 winter wheat. https://ragt-seeds.co.uk/en-gb/nos-varietes/rgt-skyfall-winter-wheat.
59. Smith, G.S., Johnston, C.M., and Cornforth, I.S. (1983). Comparison of nutrient solutions for growth of plants in sand culture. New Phytol. *94*, 537–548.
60. Babikova, Z., Gilbert, L., Bruce, T.J.A., Birkett, M., Caulfield, J.C., Woodcock, C., Pickett, J.A., and Johnson, D. (2013). Underground signals carried through common mycelial networks warn neighbouring plants of aphid attack. Ecol. Lett. *16*, 835–843.
61. van der Heijden, M.G.A., Martin, F.M., Selosse, M.-A., and Sanders, I.R. (2015). Mycorrhizal ecology and evolution: the past, the present, and the future. New Phytol. *205*, 1406–1423.
62. Declerck, S., Strullu, D.-G., and Fortin, A. (2005). In Vitro Culture of Mycorrhizas*Volume 4* (Springer Science & Business Media).
63. Blackman, R.L., and Eastop, V.F. (2000). Aphids on the World's Crops: an Identification and Information Guide (John Wiley & Sons).
64. Van Emden, H.F., and Harrington, R. (2017). Aphids as Crop Pests (Cabi).
65. Johnson, D., Leake, J.R., and Read, D.J. (2001). Novel in-growth core system enables functional studies of grassland mycorrhizal mycelial networks. New Phytol. *152*, 555–562.
66. Sun, Y.C., Chen, F.J., and Ge, F. (2009). Elevated CO_2 changes interspecific competition among three species of wheat aphids: *Sitobion avenae, Rhopalosiphum padi*, and *Schizaphis graminum*. Environ. Entomol. *38*, 26–34.
67. Vierheilig, H., Coughlan, A.P., Wyss, U., and Piché, Y. (1998). Ink and vinegar, a simple staining technique for arbuscular-mycorrhizal fungi. Appl. Environ. Microbiol. *64*, 5004–5007.
68. McGonigle, T.P., Miller, M.H., Evans, D.G., Fairchild, G.L., and Swan, J.A. (1990). A new method which gives an objective measure of colonization of roots by vesicular—arbuscular mycorrhizal fungi. New Phytol. *115*, 495–501.
69. Tennant, D. (1975). A test of a modified line intersect method of estimating root length. J. Ecol. *63*, 995–1001.
70. Cameron, D.D., Johnson, I., Leake, J.R., and Read, D.J. (2007). Mycorrhizal acquisition of inorganic phosphorus by the green-leaved terrestrial orchid *Goodyera repens*. Ann. Bot. *99*, 831–834.
71. Murphy, J., and Riley, J.P. (1962). A modified single solution method for the determination of phosphate in natural waters. Anal. Chim. Acta *27*, 31–36.
72. Cameron, D.D., Johnson, I., Read, D.J., and Leake, J.R. (2008). Giving and receiving: measuring the carbon cost of mycorrhizas in the green orchid, *Goodyera repens*. New Phytol. *180*, 176–184.

STAR★METHODS

KEY RESOURCES TABLE

REAGENT or RESOURCE	SOURCE	IDENTIFIER
Chemicals, Peptides, and Recombinant Proteins		
Sodium hypochlorite solution	N/A	N/A
Hydrochloric acid	Fisher	H/1200/PB17
Potassium Nitrate	Fisher	P/6040/60
Calcium Nitrate.4H_2O	Sigma	C1396
Sodium diHydrogen Orthophosphate.2H_2O	Fisher	S/3760/53
Magnesium Sulfate.7H_2O	Fisher	M/1000/60
EDTA - Iron III sodium salt	Sigma	EDF5
Manganese Sulfate.4H_2O	Fisher	M/2300/50
Zinc Sulfate.7H2O	SLS	CHE3938
Copper II Sulfate.5H_2O	Sigma	C3036
Boric acid	Generon	BB0044
Sodium Molybdate.2H_2O	Acros	206371000
Sodium Chloride	VWR	443824T
Potassium Chloride	Fisher	P/4240/53
Potassium Phosphate monobasic	Sigma	P0662
D-Calcium Pantothenate 98%	Acros	243300050
Biotin	Fluka	14400
Nicotinic acid	Acros	128291000
Pyridoxine	Sigma	P5669
Thiamine-HCL	Sigma	T4625
Cyanocobalamine	Acros	405925000
Phytagel	Sigma	P8169
Sucrose	Fisher	S/8560/63
^{33}P-phosphoric acid	Perkin Elmer	NEZ080
^{33}P-phosphoric acid	Hartmann Analytic	FF-01
Lactic acid 90%	Acros	189870010
^{14}C-sodium bicarbonate	Perkin Elmer	NEC086H001MC
^{14}C-sodium bicarbonate	Hartmann Analytic	ARC0138A
Potassium Hydroxide	Acros	134060010
Ethanol	Sigma	32221
Pelikan Brilliant Black ink	N/A	N/A
Acetic acid - glacial	VWR	8187552500
Poly(vinyl alcohol)	Sigma	363146
Glycerol	Acros	158920025
Trypan blue	Acros	189351000
Phenol	Fisher	BP226-100
Sulphuric acid	VWR	20700.323
Hydrogen Peroxide 35%	Acros	202460010
Emulsify-safe	Perkin Elmer	6013389
Ammonium Molybdate.4H_2O	Generon	AB0067
Ascorbic acid	Sigma	A92902
Sodium Hydroxide	Fisher	BP359-500
Sodium diHydrogen Orthophosphate.2H_2O	Fisher	S/3760/53

(*Continued on next page*)

Continued		
REAGENT or RESOURCE	SOURCE	IDENTIFIER
CarbonTrap	Meridian Biotechnologies	CT/10
CarbonCount	Meridian Biotechnologies	CC/10
Permafluor® E+	Perkin Elmer	6013187
Carbo-Sorb® E	Perkin Elmer	6013729
Experimental Models: Organisms/Strains		
Triticum aestivum L., cv. Skyfall	RAGT Seeds Ltd.	N/A
Rhizophagus irregularis Schenck and Smith isolate 09	N/A	N/A
Rhopalosiphum padi	Dr. Tom Pope, Harper Adams University	N/A
Software and Algorithms		
GraphPad Prism v8.2.0	GraphPad Software	https://graphpad.com
R v3.6.2	R	http://R-project.org
R Studio v1.1.453	RStudio, Inc	https://rstudio.com
R: e1071	N/A	https://cran.r-project.org/web/packages/e1071/index.html
R: lsmeans	N/A	https://cran.r-project.org/web/packages/lsmeans/index.html
R: multcompView	N/A	https://cran.r-project.org/web/packages/multcompView/index.html

LEAD CONTACT AND MATERIALS AVAILABILITY

Further information and requests for resources, reagents, datasets, and protocols should be directed to and will be fulfilled by the Lead Contact, Professor Katie Field (k.field@leeds.ac.uk).

EXPERIMENTAL MODEL AND SUBJECT DETAILS

Plant material and growth conditions

Seeds of *Triticum aestivum* (L.) were provided by RAGT Seeds Ltd. (Saffron Walden, UK). cv. Skyfall (see Key Resources Table) was selected given its standing as the most extensively grown winter wheat variety in the UK [58]. Seeds were surface-sterilized inside a desiccator for 3 hr with chlorine gas liberated from 100 mL sodium hypochlorite with 3 mL HCl. Seeds were germinated at 20°C for 6 days in 9 cm Petri dishes on sterile filter paper (Whatman No 1., Watman plc., Kent, UK) moistened with 4 mL autoclaved dH_2O. Two germinated seedlings were planted in 4.5'' pots in substrate consisting of a pre-sterilized sand: perlite mix (3:1), inoculated with the AM fungus *Rhizophagus irregularis* (see Fungal material and culture conditions). Seedlings were later thinned to one plant per pot after 14-days growth (48, n = 12). Pot surfaces were covered with 3 mm HDPE pellets (Northern Polymers & Plastics Ltd., Cheshire, UK) to stop algal growth and prevent water loss.

Plants were grown inside insect rearing tents (BugDorm 44545, Watkins & Doncaster, Herefordshire, UK) in controlled environment growth cabinets (Snijder Microclima 1000, Tilburg, Holland) at the University of Leeds. Growth conditions were kept at 20°C and 70% relative humidity (RH) throughout a 16-hr day-time cycle, during which LED light intensities averaged 210 μmol m^{-2} s^{-1} at canopy level. Environmental conditions during the 8-hr night-time cycle were 15°C and 70% RH. Atmospheric CO_2 concentrations were maintained at 440 ppm ('aCO_2') or 800 ppm ('eCO_2'). Plants were fed once a week with 30 mL low-P (40%) nitrate-type Long Ashton Solution (LAS) [59]. Feeding frequencies were increased to twice a week between weeks 4 and 6, beyond which nutrient strengths were reduced (20%). Plants not exposed ('- aphids') and exposed ('+ aphids') to aphid herbivores (see Aphid material and culture conditions) were grown at different times (- aphids: 5th April 2017 – 14th June 2017; + aphids: 24th July 2017 – 4th October 2017) to control for any potential impact of herbivore-induced plant volatiles on plant-AM fungal resource exchange [60]. Likewise, plants were switched between cabinets every month to control for any growth cabinet effect.

Fungal material and culture conditions

All plants were inoculated with a single AM fungal isolate of *Rhizophagus irregularis* (see Key Resources Table). *R. irregularis* was selected given its generalist host-range [61], global distribution [32], and cooperative function [18]. *In vitro* cultures of the AM fungus were grown on transformed carrot (*Daucus carota* L.) root in 20 cm Petri dishes on Phytagel MSR medium [62]. Cultures were incubated in the dark at 22°C (Sanyo MIR-553, Cardiff, UK). 6 plates dated between 24th June 2016 and 7th July 2016 were blended using a counter-top processor (Philips HR2162/91, Drackten, Holland) and diluted with autoclaved dH_2O. Spore counts were conducted in

triplicate using 100 μL of inoculum with a compound microscope (L1500, GX Microscopes, Sudbury, UK). 15 mL of inoculum, consisting of approximately 12,900 *R. irregularis* spores, was mixed evenly through the substrate added to each pot.

Aphid material and culture conditions

Rhopalosiphum padi aphids were kindly gifted by Dr. Tom Pope, Harper Adams University (see Key Resources Table). The bird cherry-oat aphid was selected given its specialist host-range [63] and status as the main pest of cereals in temperate agro-ecosystems [64]. Cultures of *R. padi* were reared on winter wheat (*T. aestivum*, L.) inside insect rearing tents in semi-controlled glasshouse conditions at the University of Leeds. Plants were grown in composted soil at 20°C and watered twice a week. Light intensities averaged 150 $\mu mol\ m^{-2}\ s^{-1}$ during a 16-hr-light/8-hr-dark photoperiod under high pressure sodium lamps. Aphids subsequently introduced to plants grown at eCO_2 were not acclimated to high [CO_2] prior to exposure to experimental plants.

METHOD DETAILS

Experimental set-up

At the time of planting, three windowed PVC cores lined with 35 μm nylon mesh (PlastOk Ltd., Birkenhead, UK) were inserted into the pot substrate (Figure S1A). Mesh, affixed to sides and base of the cores using Tensol® 12 acrylic adhesive (Bostik Ltd, Staffordshire, UK), excluded roots of cv. Skyfall plants but permitted access of extra-radical fungal hyphae [65]. Two of the cores were filled with bulk substrate (99.25% core volume) and fine-ground tertiary basalt (0.75% core volume) that acted as fungal bait [22]. A silicone capillary tube (Smith Medical Inc., Kent, UK) was attached centrally to these cores, via which ^{33}P was later introduced in an aqueous solution to one core in each pot (see 33P isotope tracing). The third core was filled with glass wool (Acros Organics, Geel, Belgium) and fitted with a Suba-Seal® rubber septum (Sigma-Aldrich, Darmstadt, Germany). This core allowed for the sampling of below-ground respiration and flux of ^{14}C by the extra-radical mycelium of the AM fungus throughout the ^{14}C labeling period (see 14C label).

Aphid exposure

After 8 weeks growth, one insect clip cage was secured to the third leaf on the main tiller of each plant (Figure S1B). Half of all replicates (n = 24) were exposed to five apterous *Rhopalosiphum padi* aphids transferred from culture plants using a paint brush. Insect clip cages were suspended above the substrate surface so as not to separate the leaf from the plant.

As growth rates of *R. padi* can respond positively to elevated atmospheric [CO_2] [66], aphid abundance in each clip cage was recorded every 24-48 hr during the subsequent dual-isotope labeling period (see 33P isotope tracing and ^{14}C label). Final aphid abundance counts were conducted prior to the ^{14}C pulse. While *R. padi* growth rates were greater at eCO_2 than at aCO_2 (Figure S3A), final aphid abundance - which were used for the measurement of aphid-acquired C - were not significantly different (Figure S3B). Moreover, assimilation of recently-fixed plant C by aphids was equivalent across [CO_2] treatments when expressed as total aphid C (Figure S3C) or as a % of plant-fixed C (Figure S3D), meaning the external biotic C sink strengths were the same under contrasting [CO_2]. Therefore, not exposed (- aphids) and exposed (+ aphids) was included in the statistical model as a categorical explanatory variable (see Data analyses).

^{33}P isotope tracing

24 hr after insect clip cages were positioned on plants, a 100 μL aqueous solution containing 1 MBq ^{33}P-orthophosphate (- aphids: 5.76 TBq mg^{-1} SA, 0.17 ng; + aphids: 3.12 TBq mg^{-1} SA, 0.32 ng) was introduced directly into one of the mesh-walled cores in each pot via the capillary tube fitted centrally (Figure 1A). Tubing had been pierced using a mounted needle every 0.5 cm below the substrate surface, ensuring an even distribution of isotope solution through the core substrate. Cores to which isotope tracer was added were rotated in half of all of the experimental pots (n = 6, 'rotated' treatment), breaking hyphal connectivity between plants and the core substrate. Core rotation was performed prior to the addition of ^{33}P and every 48 hr thereafter. The second substrate-filled core in these pots was kept static, which preserved hyphal connectivity between wheat and the core. In the remaining half of the pots (n = 6, 'static' treatment), labeled cores were not rotated and therefore plants maintained hyphal connections with the mesh-walled core. Non-labeled cores within these replicates were rotated, controlling for hyphal disturbance and effects on mass flow. By subtracting plant-assimilated ^{33}P within the 'rotated' treatment from the 'static' treatment, the movement of isotopes out of the cores by diffusion or alternative microbial nutrient cycling processes and into plants was controlled for [22].

^{14}C label

12 days after labeling with ^{33}P, the tops of both substrate cores were sealed using vial caps and anhydrous lanolin, and pots were enclosed in airtight chambers (Polybags Ltd, London, UK) (Figure 1B). A 1.036-MBq pulse of $^{14}CO_2$ gas was liberated into the headspace of plants at the beginning of the 16-hr photoperiod, by adding 2 mL 10% lactic acid to a cuvette containing 28 μL ^{14}C-sodium bicarbonate (- aphids: 1620.6 MBq $mmol^{-1}$ SA; + aphids: 1850 MBq $mmol^{-1}$ SA). Cuvettes were attached to plant labels implanted in the substrate next to the base of each plant. 1 mL of labeled headspace gas was sampled immediately using a hypodermic syringe and 1.5 and 4.5 hr later, which recorded the drawdown of $^{14}CO_2$ by plants. Below-ground gas samples were taken via the glass-wool core immediately following the liberation of ^{14}C and every 90 mins thereafter, measuring respiration and flux of $^{14}CO_2$ by the AM fungal network. Above- and below-ground gas samples were injected into separate gas-evacuated 20 mL scintillation vials containing equal volumes (i.e., 10 mL) of the liquid scintillants Carbo-Sorb® and Permafluor® (Perkin Elmer, Beaconsfield, UK). Sample

radioactivity was quantified by liquid scintillation counting (Tri-Carb® 3100TR, Perkin Elmer, Beaconsfield, UK). At the end of the 16-hr photoperiod, 4 mL 2M KOH was injected into vial caps inside each airtight chamber to capture remaining $^{14}CO_2$ gas before plants were harvested.

Plant harvest and sample preparation

Insect clip cages were removed from all plants and aphids on '+ aphid' replicates stored at −20°C. Mesh-walled cores were extracted from the substrate and pots were separated into shoots, roots, bulk substrate, rotated core substrate, and static core substrate. Roots were cleaned with tap water and a sub-sample taken for quantification of AM root length colonization, being stored in 50% EtOH (v/v) at 5°C. 10-15 g of bulk substrate was also stored at 5°C for quantification of AM hyphal lengths. Remaining plant and substrate material were stored at −20°C for 24 hr and freeze-dried with aphid samples for 3 days (CoolSafe 55-4, LaboGene, Allerød, Denmark). Dry weight measurements of each component were taken using a 5-digit digital scale (Quintix 224-1S, Satorious Lab Instruments, Goettingen, Germany), before being analyzed for P, ^{33}P and ^{14}C.

AM fungal colonization of roots and bulk substrates

Root samples were cleared in 10% KOH (w/v) at 80°C for 40 mins and AM fungal structures stained with ink and vinegar stain (5% Pelikan Brilliant Black, 5% acetic acid, 90% dH_2O) [67]. Roots were de-stained in 1% acetic acid and mounted on microscope slides using polyvinyl lacto-glycerol (16.6 g polyvinyl alcohol powder, 10 mL glycerol, 100 mL lactic acid, 100 mL dH_2O). Assessments of % root length colonization, % arbuscules, and % vesicles were made using the magnified intersection methodology (150 intersects per plant, 400x magnification) [68].

AM fungal hyphae were extracted from 4-5 g of bulk substrate in 500 mL dH_20, from which 10 mL was filtered through two 0.45 μm membrane filters (Watman plc., Kent, UK) and stained with Trypan Blue solution (0.4 g Trypan Blue stain, 20% phenol, 20% lactic acid, 20% dH_2O, 40% glycerol). AM hyphal lengths per pot were calculated using the gridline-intersection methodology (50 fields of view, 100x magnification) [69].

Plant- and mycorrhizal-acquired P and ^{33}P

Freeze-dried plant material was homogenized using a mill (A10 Basic, IKA®, Oxfordshire, UK). 30-40 mg of shoot, root, and substrate sample were digested in triplicate in 1 mL concentrated sulphuric acid at 365°C for 15 mins. 100 μL of hydrogen peroxide was added to cooled samples and returned to the digest block (Grant BT5D, Cambridgeshire, UK). Cleared digest solutions were then diluted to 10 mL with dH_2O. ^{33}P-radioactivity of plant and substrate material was quantified through liquid scintillation (Tri-Carb® 3100TR Perkin Elmer, Beaconsfield, UK). 2 mL of each digest solution was added to 10 mL of Emulsify-safe scintillant, and ^{33}P content was calculated using Equation 1 [70].

$$M^{33}P = \left(\left[\frac{\frac{cDPM}{60}}{SAct}\right]\right)MwtDF$$

Equation 1. Where $M^{33}p$ = mass of ^{33}P (mg); cDPM = counts as disintegrations per min; Sact = specific activity of the course (Bq $mmol^{-1}$); Df = dilution factor; and Mwt = molecular mass of P.

Total P content of plant material was determined using an adapted method from [71]. 0.15 mL and 0.2 mL of shoot and root digest solutions were added to separate cuvettes with 0.5 mL ammonium molybdate, 0.2 mL of 0.1 M L-ascorbic acid, and 0.2 mL 3.44 M sodium hydroxide. Solutions were made up to 3.8 mL with dH_20, and the optical density of samples recorded after 45 mins at 822 nm using a spectrophotometer (Jenway 6300, Staffordshire, UK). A 10 mg L^{-1} standard P solution was made by dissolving 44.55 mg of sodium dihydrogen orthophosphate in 1L dH_20, and a standard curve was produced against which total sample P was calculated (Figure S5).

Plant C transfer to the AM fungus and assimilation by aphids

^{14}C within plant, substrate, and aphid samples was quantified through sample oxidation (Model 307 Packard Sample Oxidiser, Isotech, Chesterfield, UK) and liquid scintillation (Tri-Carb® 3100TR Perkin Elmer, Beaconsfield, UK). 20-30 mg of freeze-dried shoot and root material was weighed in triplicate into Combusto-cones (Perkin Elmer, Beaconsfield, UK), as was 30-40 mg of bulk substrate, rotated core substrate, and static core substrate from each pot, and all aphids removed from each plant. ^{14}C within plant, substrate, and aphid material was released following sample oxidation (Model 307 Packard Sample Oxidiser, Isotech, Chesterfield, UK) and CO_2 trapped in 10 mL of the liquid scintillant CarbonTrap and mixed with 10 mL CarbonCount (Meridian Biotechnologies Ltd., Tadworth, UK). Radiation within samples was then quantified through liquid scintillation counting (Packard Tri-carbon 3100 TR, Isotech, Chesterfield, UK). Total C fixed by plants (i.e., $^{12}CO_2$ and $^{14}CO_2$) and transferred to the AM fungus or assimilated by aphids was calculated by determining the total CO_2 volume and content mass in the airtight chamber and proportion of $^{14}CO_2$ that was photosynthetically fixed by plants, using Equations 2-3 from [72].

$$T_{pf} \text{ or } T_{pa} = \left(\left(\frac{A}{A_{sp}}\right)m_a\right) + (P_r \times m_c)$$

Equation 2: Where T_{pf} or T_{pa} = total C transferred from plant to fungus or assimilated by aphids (g); A = radioactivity of the tissue sample (Bq); A_{sp} = specific activity of the source (Bq Mol^{-1}); m_a = atomic mass of ^{14}C; P_r = proportion of the total ^{14}C label supplied present in the tissue; and m_c = mass of C (g) in the CO_2 present in the labeling chamber, from the ideal gas law (Equation 3).

$$m_{cd} = M_{cd}\left(\frac{PVcd}{RT}\right) \therefore m_c = m_{cd} \times 0.27292$$

Equation 3: Where m_{cd} = mass of CO_2 (g); M_{cd} = molecular mass of CO2 (44.01 g mol^{-1}); p = pressure (kPa); V_{cd} = volume of CO_2 in the chamber (0.003 m^3); m_c = mass of unlabelled C in the labeling chamber (g); M = Molar mass (12.011 g); R = universal gas constant (J K^{-1} mol^{-1}); T = absolute temperature (K); m_c = mass of C (g) in the CO_2 present in the labeling chamber, where 0.27292 is the proportion of C in CO_2 on a mass fraction basis.

The difference between ^{14}C recovered in the substrate of rotated and static cores in each pot is representative of recently-fixed plant C transferred to the extraradical mycelium of the AM fungus. Rotated core values provided an internal control for the movement of ^{14}C into the core via diffusion (i.e., of dissolved C in the bulk substrate from root respiration and/or exudation) or through alternative microbial C cycling processes [48].

QUANTIFICATION AND STATISTICAL ANALYSIS

Data analyses

All statistical analyses were performed in R Studio v1.1.453. Data were tested for normality and homogeneity of variances using normal probability plots and residuals versus fits plots. The effects of aphid herbivory, [CO_2], and their interaction on shoot biomass, shoot ^{33}P, shoot [^{33}P], and shoot [C] were analyzed using a generalized linear model (GLM) and additional post hoc Tukey honest significant difference (HSD) tests. Root biomass, hyphal lengths, shoot P and [P], root P and [P], root [C], aphid growth rates, final aphid abundance, aphid C, and % plant-fixed C assimilated by aphids and allocated to the static core were Log_{10} transformed and then analyzed using GLM. Root ^{33}P, root [^{33}P], and AM fungal network ^{33}P were square root transformed, and % root length colonization, % arbuscules, and % vesicles were arcsine square root transformed before being analyzed using GLM. Fungal C could not be transformed to meet parametric test assumptions and so was analyzed using Mann-Whitney U. Spearman's rank correlation coefficients were performed between shoot ^{33}P and % root length colonization and plant C allocation to the AM fungus. All figures were produced using GraphPad Prism v.8.2.0.

DATA AND CODE AVAILABILITY

The datasets generated during this study are available at Mendeley Data, https://doi.org/10.17632/dwm2ttb5rv.1. Data are also available on request from the lead author.

Current Biology, Volume *30*

Supplemental Information

Aphid Herbivory Drives Asymmetry in Carbon for Nutrient Exchange between Plants and an Arbuscular Mycorrhizal Fungus

Michael D. Charters, Steven M. Sait, and Katie J. Field

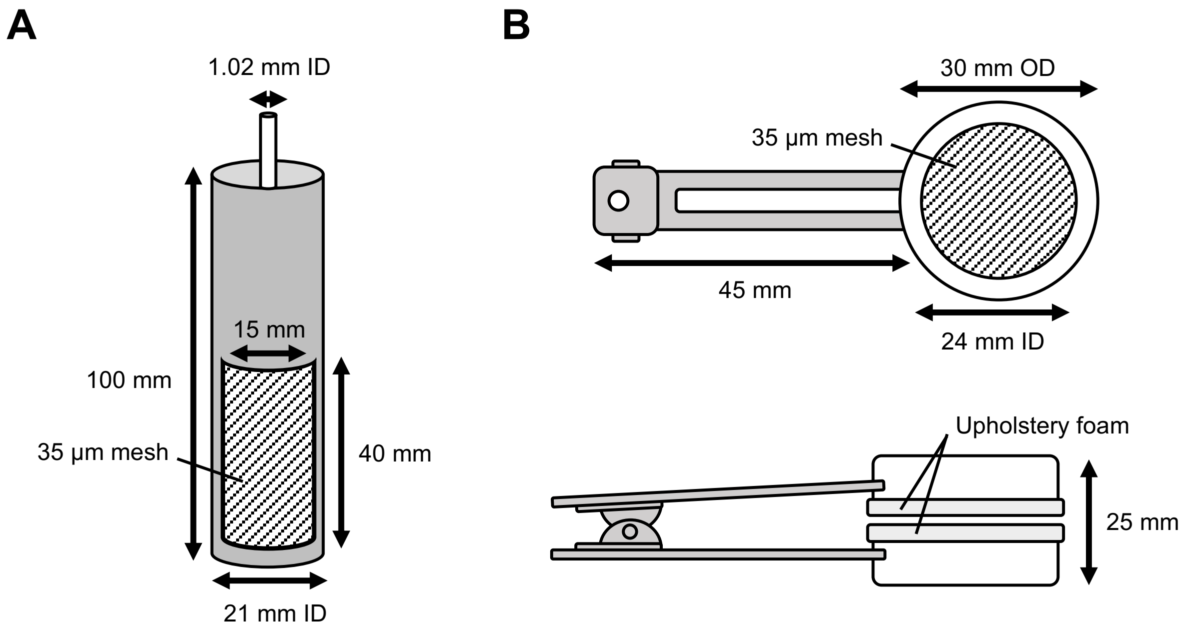

Figure S1. Schematic diagrams of PVC cores and insect clips cages. 'ID' and 'OD' refer to inner and outer diameter measurements. Related to Figure 1 and STAR METHODS.

(A) Dimensions of the three PVC cores added to the substrate of each pot. Two windows were machine-cut into opposite faces of the core, and covered with 35 µm nylon mesh using an acrylic adhesive. Mesh apertures permitted in-growth of AM fungal hyphae but not plant roots. A silicone capillary tube was perforated and attached centrally. This acted as a conduit for the introduction of ^{33}P to the two substrate-filled cores. The third core contained glass wool and had a rubber septum fitted on top. **(B)** Dimensions of the insect clip cages positioned on the third leaf of the primary tiller of plants. Cages were constructed using machine-cut transparent acrylic tubing and double prong curl setting clips. Upholstery foam and 35 µm nylon mesh were attached to the inside and outside faces of the cage using an acrylic adhesive, which limited damage to the leaf surface and kept aphids confined.

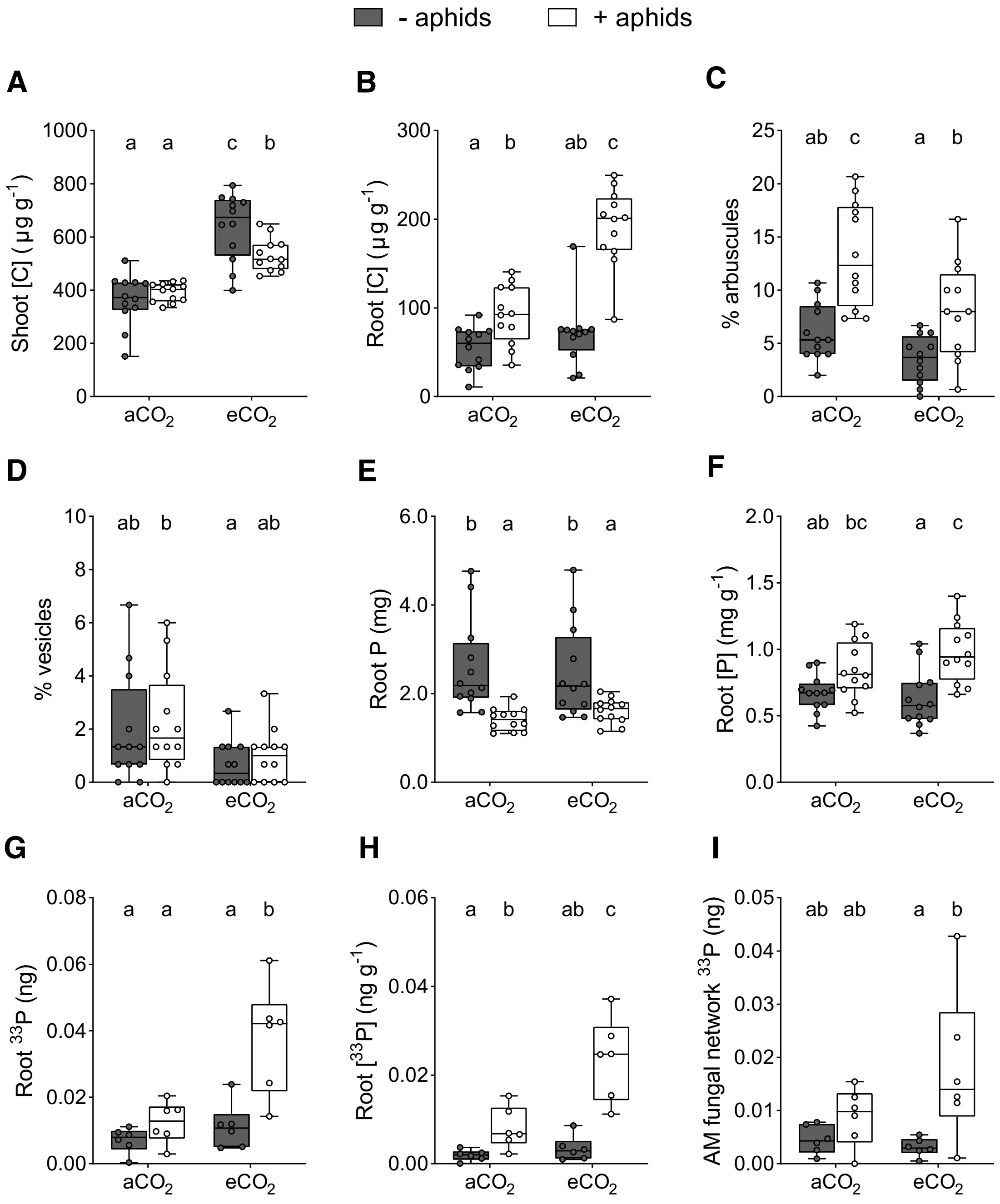

Figure S2. Supplementary data for traits in plants not exposed (gray boxes) or exposed (white boxes) to aphid herbivores at ambient and elevated atmospheric [CO_2]. Related to Figures 2, 3, and 5.

(A) Shoot C concentration. **(B)** Root C concentration. **(C)** % arbuscular colonization. **(D)** % vesicular colonization. **(E)** Root P content. **(F)** Root P concentration. **(G)** Root ^{33}P content. **(H)** Root ^{33}P concentration. **(I)** ^{33}P in the AM fungal hyphal network. Boxplots extend from the first to the third quartile, with the middle line representing median values (n = 12, except for G, H, and I where n = 6). Whiskers are drawn to the minimum and maximum data points (open or closed markers). Different letters denote significant differences between treatment means (where $p < 0.05$, GLM+Tukey HSD tests).

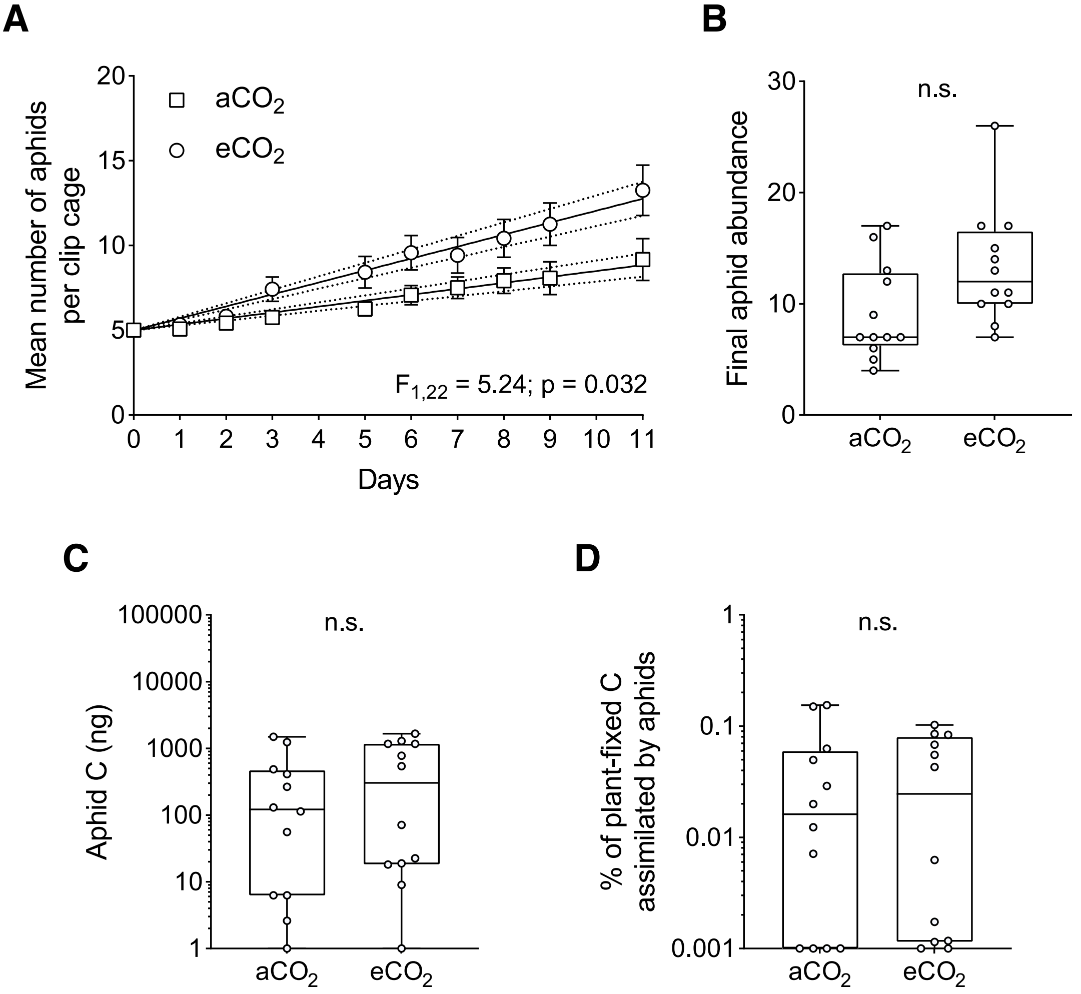

Figure S3. Effect of atmospheric [CO_2] on wheat-feeding aphids (*Rhopalosiphum padi*). Related to Figure 4 and STAR METHODS.

(A) Aphid population growth rates in insect clip cages on 8-week old plants grown at aCO_2 (440 ppm; circular markers) and eCO_2 (800 ppm; square markers) during the 12-day isotope labelling period (n = 12; mean ± SE). Dashed lines represent 95% confidence intervals. **(B)** Final aphid abundance inside insect clip cages prior to the $^{14}CO_2$ pulse. **(C)** Assimilation of recently fixed plant C by aphids (log scale). **(D)** % of plant-fixed C assimilated by aphids (log scale). Boxplots extend from the first to the third quartile, with the middle line representing median values (n = 12). Whiskers are drawn to the minimum and maximum data points (open markers). 'n.s.' denotes no significant difference between treatments, as final aphid abundance ($F_{1,22} = 3.73$; $p = 0.066$), aphid C ($F_{1,22} = 0.23$; $p = 0.636$), and the % of plant-fixed C assimilated by aphids ($F_{1,22} = 0.01$; $p = 0.961$) were equivalent at aCO_2 and eCO_2.

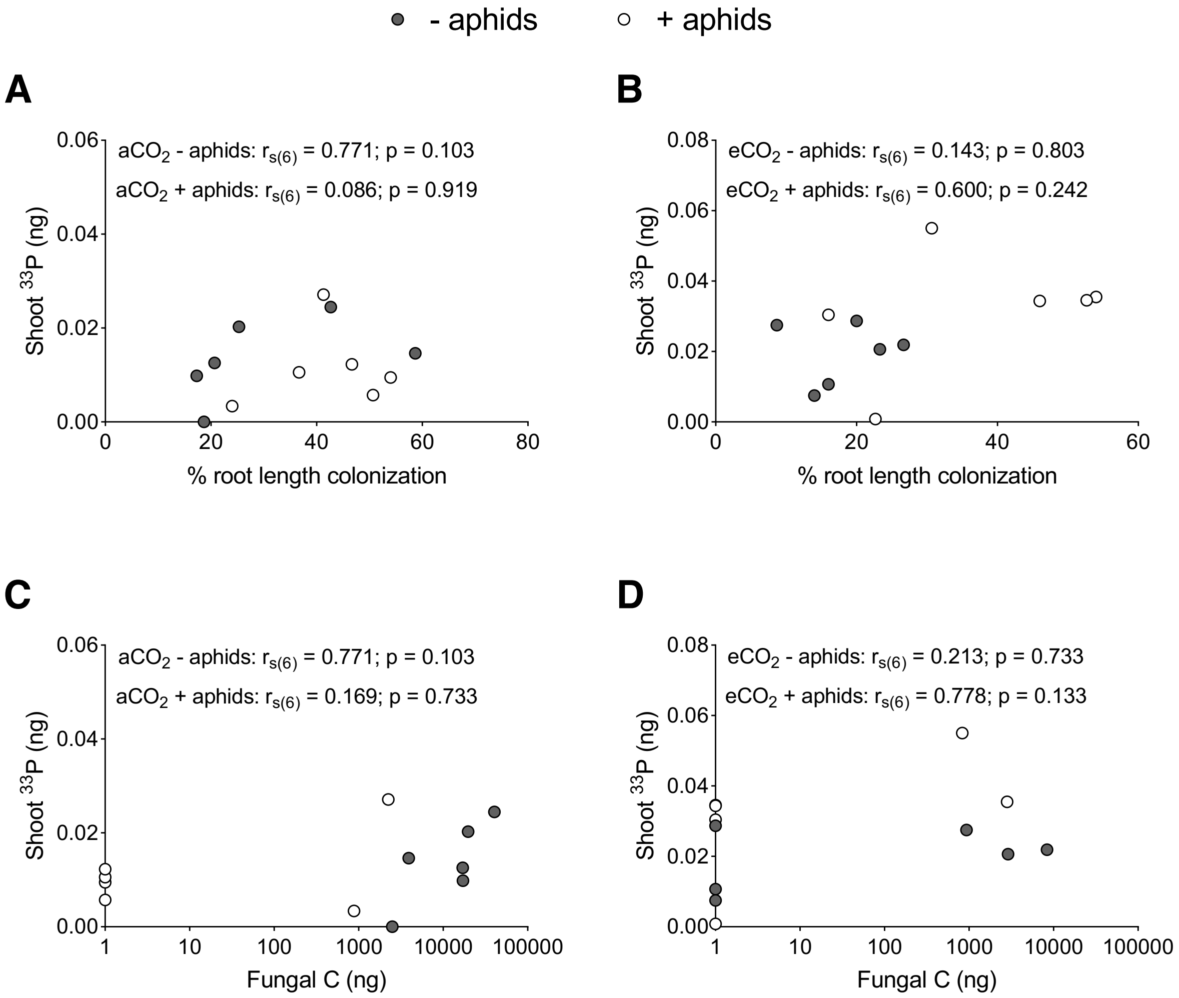

Figure S4. Mycorrhizal uptake of ^{33}P as a function of AM abundance in roots of wheat or transfer of recently-fixed plant C to the AM fungus. Related to Figures 3, 4, and 5.

(A-B) Relationship between AM fungal-acquired shoot ^{33}P and % root length colonization in plants not exposed (gray markers) and exposed (white markers) to aphid herbivores at ambient (A) and elevated (B) atmospheric [CO_2]. Associations analyzed using Spearman's rank correlation coefficient.

(C-D) Relationship between AM fungal-acquired shoot ^{33}P and plant C transfer to the AM fungus in plants not exposed (gray markers) and exposed (white markers) to aphid herbivores at ambient (C) and elevated (D) atmospheric [CO_2]. Associations analyzed using Spearman's rank correlation coefficient.

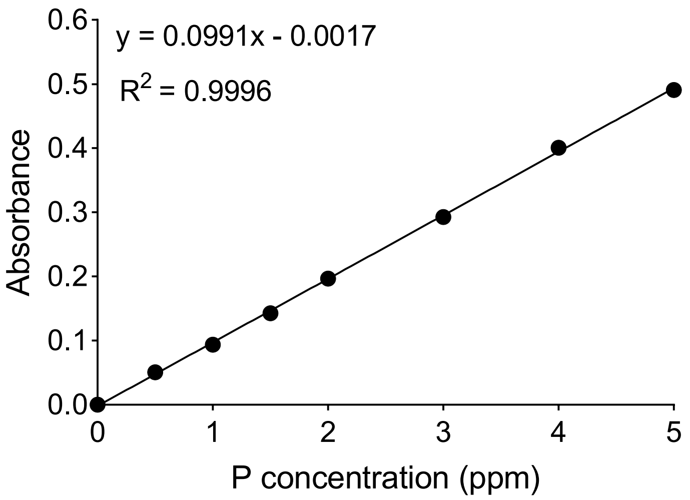

Figure S5. Calibration curve for total P determination of plant material. Related to Figure 5 and STAR METHODS.

Response variable	Factor	F-value	df	p-value
Shoot [C]	Aphids	2.40	1,44	0.128
	[CO_2]	67.36	1,44	**<0.001**
	Aphids * [CO_2]	7.13	1,44	**0.011**
Root [C]	Aphids	36.61	1,44	**<0.001**
	[CO_2]	13.92	1,44	**<0.001**
	Aphids * [CO_2]	3.37	1,44	0.073
% arbuscules	Aphids	27.03	1,44	**<0.001**
	[CO_2]	13.64	1,44	**<0.001**
	Aphids * [CO_2]	0.26	1,44	0.610
% vesicles	Aphids	1.10	1,44	0.300
	[CO_2]	8.91	1,44	**0.005**
	Aphids * [CO_2]	0.01	1,44	0.915
Root P content	Aphids	28.72	1,44	**<0.001**
	[CO_2]	0.16	1,44	0.687
	Aphids * [CO_2]	1.21	1,44	0.278
Root [P]	Aphids	21.14	1,44	**<0.001**
	[CO_2]	0.67	1,44	0.416
	Aphids * [CO_2]	2.43	1,44	0.126
Root ^{33}P content	Aphids	16.28	1,20	**<0.001**
	[CO_2]	13.38	1,20	**0.002**
	Aphids * [CO_2]	4.29	1,20	0.051
Root [^{33}P]	Aphids	47.88	1,20	**<0.001**
	[CO_2]	15.02	1,20	**<0.001**
	Aphids * [CO_2]	6.04	1,20	**0.023**
AM fungal network ^{33}P	Aphids	7.86	1,20	**0.011**
	[CO_2]	0.74	1,20	0.401
	Aphids * [CO_2]	2.41	1,20	0.136

Table S1. GLM outcomes: main treatment effects (aphid herbivory and atmospheric [CO_2]) and their interaction on response variables measured. Significant p-values ($p < 0.05$) are in bold. Related to Figures 2, 3, 5, and S2.

Response variable	Factor	U_{min}	U_{max}	$n_1 = n_2$	p-value
Fungal C	(i) aCO_2 - aphids * aCO_2 + aphids	22	122	12	**0.002**
	(ii) aCO_2 - aphids * eCO_2 - aphids	56.5	87.5	12	0.367
	(iii) aCO_2 - aphids * eCO_2 + aphids	32	112	12	**0.015**
	(iv) eCO_2 - aphids * eCO_2 + aphids	35	109	12	**0.025**
	(v) aCO_2 + aphids * eCO_2 + aphids	60	84	12	0.362
	(vi) aCO_2 + aphids * eCO_2 - aphids	25	119	12	**0.003**

Table S2. Mann-Whitney U outcomes: main treatment effects (aphid herbivory [i & iv] and atmospheric [CO_2] [ii & v]), and their interaction (iii & vi) on the transfer of recently-fixed plant C to the AM hyphal network in the pot. Significant p-values ($p < 0.05$) are in bold. Related to Figure 4A.
